# Holmium-166 Radioembolization Is a Safe and Effective Locoregional Treatment for Primary and Secondary Liver Tumors: A Systematic Review and Meta-Analysis

**DOI:** 10.3390/cancers17111841

**Published:** 2025-05-31

**Authors:** Petra Sólymos, Mátyás Rédei, Caner Turan, Bence Szabó, Alexandra Ádám, Zsolt Molnár, Gábor Duray, Péter Hegyi, Dénes B. Horváthy

**Affiliations:** 1 Centre for Translational Medicine, Semmelweis University, 1085 Budapest, Hungary; solymos.petra@semmelweis.hu (P.S.); matyas.redei@semmelweis.hu (M.R.); caner.turan@semmelweis.hu (C.T.); szabo.bence1@semmelweis.hu (B.S.); adam.alexandra@stud.semmelweis.hu (A.Á.); molnar.zsolt1@semmelweis.hu (Z.M.); duray.gabor@epc-honvedkorhaz.hu (G.D.); hegyi.peter@semmelweis.hu (P.H.); 2 Department of Radiology, Medical Imaging Centre, Semmelweis University, 1082 Budapest, Hungary; 3 Department of Anesthesiology and Intensive Therapy, Semmelweis University, 1082 Budapest, Hungary; 4 Institute for Translational Medicine, Medical School, University of Pécs, 7624 Pécs, Hungary; 5 Department of Anesthesiology and Intensive Therapy, Poznan University of Medical Sciences, 61701 Poznan, Poland; 6 Department of Cardiology, Central Hospital of Northern Pest—Military Hospital, 1134 Budapest, Hungary; 7 Institute of Pancreatic Diseases, Semmelweis University, 1083 Budapest, Hungary; 8 Department of Interventional Radiology, Heart and Vascular Centre, Semmelweis University, 1122 Budapest, Hungary

**Keywords:** holmium-166, transarterial radioembolization, TARE, SIRT, liver tumor

## Abstract

Background/Objectives: This systematic review and meta-analysis evaluated the effectiveness and the safety of transarterial radioembolization using Holmium-166 microspheres (Ho-166-TARE) for the treatment of primary and secondary liver tumors. The aim of the study was to offer a detailed analysis of clinical outcomes and the potential benefits of this innovative therapy. Methods: The study was conducted according to the PRISMA 2020 guidelines. The systematic search was performed in five databases in November 2023 and updated in June 2024. All 16 eligible studies were original research that evaluated Ho-166-TARE. The endpoints analyzed were disease control rate (DCR), overall survival (OS), progression-free survival (PFS), clinical and laboratory adverse events, healthy-liver- and tumor-liver-absorbed doses. The risk of bias was assessed using the MINORS checklist. Results: The pooled overall disease control rate (DCR) was 72% (95% CI, 46–89%); by mRECIST, it was 93% (95% CI, 71–99%); and by RECIST 1.1, it was 54% (95% CI, 22–83%) at 3-month follow-up. Overall survival (OS) at 3, 6, 12, and 30 months was 98%, 89%, 74%, and 39%, respectively. Severe clinical adverse events were minimal, although some patients showed elevated GGT levels and lymphocytopenia. Tumor-absorbed doses were nearly three times higher than those in healthy liver tissue. Conclusions: These findings suggest that Ho-166-TARE is a safe and effective locoregional treatment option for liver tumors, especially in cases where systemic therapy alone is insufficient or surgical resection is not feasible. Further studies are needed to investigate tumor-specific response, optimize dosimetry strategies, and establish standardized protocols for long-term outcome assessment.

## Simple Summary

Liver tumors are frequently diagnosed at advanced stages, which limits the availability of curative treatment options such as surgery. Transarterial radioembolization with Holmium-166 microspheres (Ho-166-TARE) is an innovative, potentially curative locoregional treatment option that delivers targeted radiation directly to liver tumors while enabling precise imaging and personalized dosimetry. This systematic review and meta-analysis synthesized data from over 300 patients across multiple clinical studies to assess the safety and efficacy of Ho-166-TARE. The results demonstrated high rates of tumor control and favorable survival outcomes with minimal severe adverse events. These findings support Ho-166-TARE as a promising therapeutic option for patients with inoperable primary or secondary liver malignancies and highlight its potential for integration into liver-directed oncologic care.

## 1. Introduction

Primary and secondary liver malignancies are a significant health burden worldwide, contributing to high cancer-related mortality rates [1,2]. Although a wide range of treatment options have been developed, the only curative treatments are surgery (resection or transplantation) and ablative techniques [3]. However, due to underlying liver conditions such as fibrosis and cirrhosis, along with frequently late-stage diagnosis, these treatments are feasible in only 20–30% of cases, making other local therapies—such as transarterial chemoembolization (TACE), transarterial embolization (TAE) and transarterial radioembolization (TARE)—appealing alternatives [3,4]. Among these palliative treatment options, TARE stands out as a potentially curative approach [5,6].

Currently, three types of radioactive microspheres are commercially available. Yttrium-90 (^90^Y) is the most commonly used radionuclide and is available in both glass (marketed as TheraSphere^®^, BWXT Medical, Ottawa, ON, Canada) and resin (marketed as SIR-Spheres^®^, Sirtex Medical, Woburn, MA, USA) forms. More recently, poly-L-lactic acid microspheres containing Holmium-166 (^166^Ho, marketed as QuiremSpheres^®^, Quirem Medical B.V., Deventer, The Netherlands) have become available across Europe, offering an alternative to Yttrium-90 microspheres with imaging features that may offer valuable benefits [7,8]. Holmium-166 microspheres exert their therapeutic effect by emitting high-energy beta radiation that induces DNA fragmentation and leads to tumor cell death [9]. In addition, they emit gamma photons, which can be utilized for SPECT imaging [7,9]. Therefore, extrahepatic deposition in gastrointestinal organs, lung shunting, and the precise quantification of the tumor dose absorbed can be assessed more accurately [10,11]. Holmium-166 also has paramagnetic properties, allowing it to be visualized by magnetic resonance imaging (MRI), which offers a higher spatial resolution than SPECT. This allows a more detailed assessment of its distribution within the liver [9,10]. Another advantage of Holmium-166 is that it can use the same microspheres for both planning and treatment procedures [8]. For treatment planning, Holmium-166-radioembolization (Ho-166-TARE) uses Holmium-166 scout, a small amount of the same microspheres that visualizes and predicts the distribution of the therapeutic dose in the liver, improving targeting and optimizing treatment plans [7,9,12]. This approach reduces variability and potential discrepancies between planning and treatment [11]. Moreover, Holmium-166 scout has demonstrated superior predictive accuracy for intrahepatic distribution compared to the commonly used Technetium-99m macroaggregated albumin (^99m^Tc-MAA) [3,11].

Overall, Holmium-166 offers potentially better imaging properties while also being an effective alternative to Yttrium-90 for the TARE of liver tumors. The clinical value of TARE, particularly with Yttrium-90, has been established in both intermediate and advanced hepatocellular carcinoma (HCC). Meta-analyses have shown comparable survival outcomes to TACE, with better tolerance and delayed progression in some cases [13,14]. These results provide a strong rationale for investigating Holmium-166 (^166^Ho) as an alternative, especially given its added imaging and dosimetry advantages. Even so, evidence on the safety and efficacy of Ho-166-TARE is limited and requires synthesis and contextualization.

This study aims to conduct a systematic review and meta-analysis of the available literature, providing a comprehensive evaluation of the efficacy and safety of transarterial radioembolization with Holmium-166 microspheres in the treatment of liver tumors.

## 2. Materials and Methods

### 2.1. Study Registration

We conducted our systematic review and meta-analysis in line with the PRISMA 2020 guidelines [15] and followed the recommendations of the Cochrane Handbook for Systematic Reviews of Interventions [16]. Our study protocol was prospectively registered in the PROSPERO database (registration number CRD42023472899).

### 2.2. Search Strategy

Our systematic search was initially conducted on 1 November 2023 in five databases: MEDLINE via PubMed, Embase, the Cochrane Central Register of Controlled Trials (CENTRAL), Scopus, and the Web of Science databases. We applied no filters or restrictions on publication date, language, or article type. To identify any newly eligible literature, we repeated the search on 5 June 2024. The following search key was used in the systematic search: (liver* OR “hepatic” OR (“liver” AND tumo*) OR (“liver” AND neoplasm*) OR (“liver” AND “cancer”) OR (“hepatocellular” AND “carcinoma”) OR “hepatocarcinoma” OR (“hepatic” AND “cancer”) OR “HCC” OR (“cholangiocellular” AND “carcinoma”) OR cholangiocarcinoma* OR “ICC” OR (“liver” AND metasta*)) AND (“TARE” OR radioemboli* OR “transarterial” OR “transarterially” OR (“transarterial” AND radioemboli*)) AND (“holmium” OR (“ho” AND “166”) OR “166ho”) (Supplementary Material S8). We also reviewed the references of the selected articles to identify additional studies that could be included in the selection process.

### 2.3. Eligibility Criteria

This meta-analysis included both randomized and non-randomized studies with original research data. Eligibility criteria were based on the research question framework. The research question was formulated by using a modified Population, Exposure, Comparator, and Outcomes (PECO) framework, where a comparator group was not sought for eligibility. Therefore, in our study, we used the PEO framework with the following criteria: population (P): adult patients (aged 18 or older) of both sexes with primary or secondary liver tumors undergoing Ho-166-TARE; exposure (E): Ho-166-TARE for the local treatment of liver tumors; main outcome (O_1_): the primary endpoint was disease control rate (DCR), defined as patients with either stable disease or objective response at 3 months according to Response Evaluation Criteria in Solid Tumors (RECIST) 1.1 [17,18,19] and Modified RECIST (mRECIST) [20]; secondary outcome (O_2_): overall survival (OS), progression-free survival (PFS), clinical and laboratory adverse events assessed according to the Common Terminology Criteria for Adverse Events (CTCAE) (version 3.0, 4.0, 4.03 and 5.0) [21,22,23,24] and tumor- and healthy-liver-absorbed dose.

Studies were eligible regardless of whether they included patients with primary or secondary liver tumors. As stated, both tumor types were included in our analysis. Since the majority of studies did not report outcomes separately by tumor origin, we pooled these data for the meta-analysis.

We excluded studies on pediatric patients and animal studies. Publications in which the study population may have overlapped with other publications were not eligible for inclusion. Reviews, meta-analyses, case reports, case series, non-peer-reviewed studies, and conference abstracts were also excluded.

### 2.4. Selection Process

The selection process for both the initial and second searches was conducted by two independent reviewers (PS and MR). The method used in the selection process remained consistent across both searches. Duplicates were identified and removed through both manual checks and automated searches using EndNote 21 software (Clarivate Analytics, Philadelphia, PA, USA). The reviewers then evaluated the remaining studies for inclusion using Rayyan (https://www.rayyan.ai, accessed in November 2023 and June 2024) and EndNote 21 software, initially by title and abstract, followed by a full-text review [25]. Any disagreements between the two reviewers were resolved by a third independent reviewer (CT). For studies involving the same patient populations, the reviewers selected only the study with the larger patient sample for inclusion.

We contacted all corresponding authors via email to request individual patient data; however, these data were not utilized due to the limited number of responses received.

### 2.5. Data Extraction

Data were independently collected from the eligible articles by two authors (P.S. and A.Á.) using a standardized data collection sheet (Microsoft Excel, Microsoft Corporation, Redmond, WA, USA, 2021). Any disagreements were resolved through discussion between the authors. The following data were extracted: (1) study characteristics: first author, the year of publication, Digital Object Identifier (DOI), study design, study population (number, age, and sex), study period, study country and centers, previous liver treatments, tumor percent and/or burden, primary tumors; (2) tumor response: DCR according to RECIST 1.1 and mRECIST at 3 months; (3) overall survival (OS) and progression-free survival (PFS); (4) clinical and laboratory adverse events after Ho-166-TARE according to CTCAE; (5) tumor-absorbed dose and healthy liver-absorbed dose.

When written data were not available, estimates were obtained from visual sources using the WebPlotDigitizer software (version 5.0).

### 2.6. Study Risk of Bias Assessment

Two authors (P.S. and A.Á.) independently conducted a risk of bias assessment following the guidelines provided by the Cochrane Handbook [2]. Disagreements were resolved through deliberation between the authors. The Methodological Index for Non-Randomized Studies (MINORS) [26] was used to assess the risk of bias and the applicability of primary studies. As we only included single-armed studies, the risk of bias was assessed in eight distinct domains, including the clearly stated aim, inclusion of consecutive patients, prospective collection of data, endpoints appropriate to the aim of the study, unbiased assessment of the study endpoint, the follow-up period appropriate to the aim of the study, loss to follow-up less than 5%, and prospective calculation of study size.

### 2.7. Statistical Analysis

Meta-analysis of single-arm studies was conducted using random-effects models to account for variability across studies. The analyses focused on several outcomes, including DCR-s, complication rates, survival probabilities, doses absorbed, and quality of life. All statistical analyses were made with R (version 4.4.1.) using the meta package for basic meta-analysis calculations and plots and the dmetar package for additional influential analysis calculations and plots [27,28,29].

## 3. Results

### 3.1. Search and Selection

**Figure 1:**
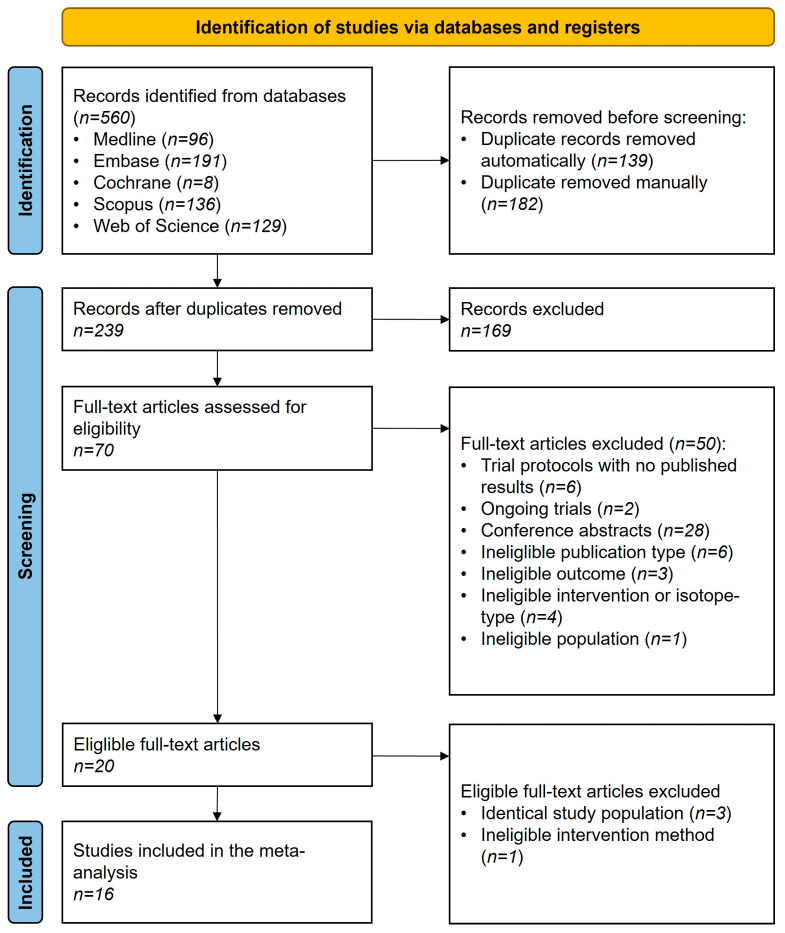
PRISMA flowchart of selection describing the systematic search and selection process.

The updated systematic search (Figure 1) yielded 560 records, and 20 eligible articles were included [30,31,32,33,34,35,36,37,38,39,40,41,42,43,44,45,46,47,48,49]. Four articles were not included in the data synthesis due to overlapping patient populations [40,41,43,46]. However, the findings are discussed as part of the systematic review. In total, 16 articles were included [30,31,32,33,34,35,36,37,38,39,42,44,45,47,48,49] in our study.

The inter-reviewer agreement was assessed using Cohen’s Kappa, which was κ = 0.90 for the title and abstract selection and κ = 0.95 for the full-text selection in the initial search. In the second search, Cohen’s Kappa was κ = 0.95 for the title and abstract selection and κ = 0.97 for the full-text selection. Cohen’s Kappa values above 0.80 were considered acceptable.

### 3.2. Basic Characteristics of Studies Included

Nine of the sixteen studies were identified as prospective interventional studies, two were prospective cohort studies, and five were retrospective cohort studies. Detailed baseline characteristics for the included studies are provided in Table 1.

### 3.3. Efficacy Assessment—Tumor Response

**Figure 2:**
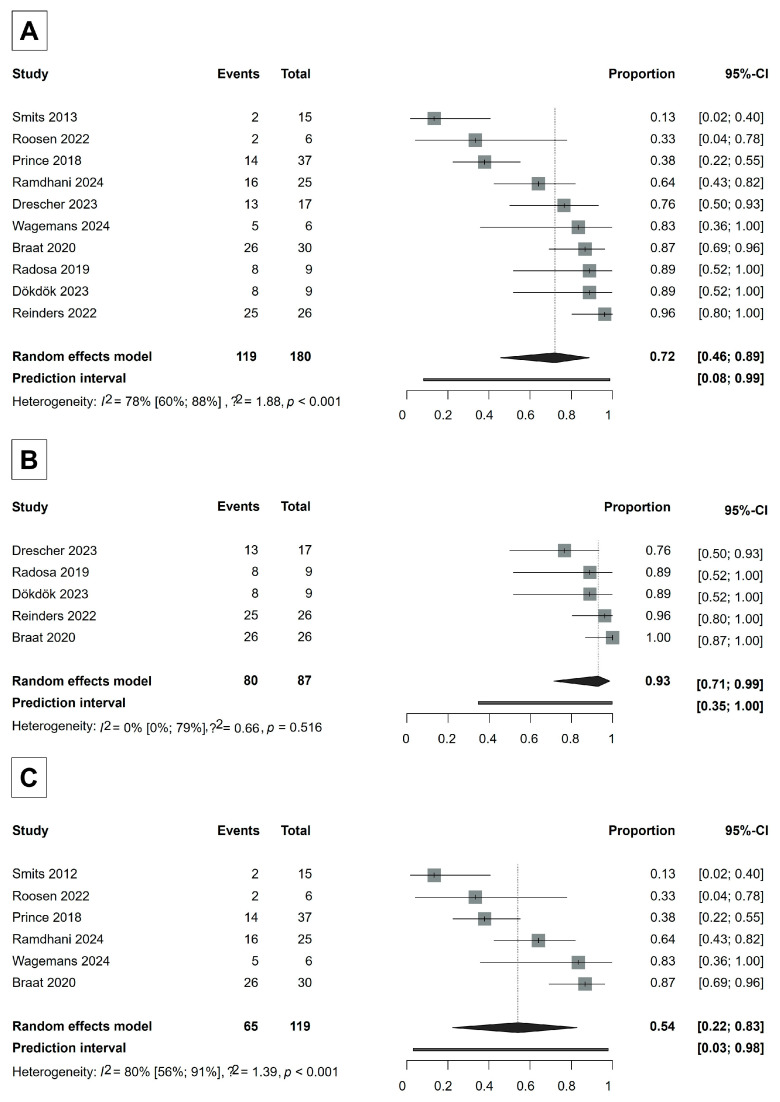
Forest plot for DCR analysis. (**A**) Overall DCR analysis. (**B**) Analysis according to mRECIST. (**C**) Analysis according to RECIST 1.1. CI = Confidence Interval. DCR = disease control rate. Proportions refer to the ratio of patients whose tumors were controlled by Ho-166-TARE [30,32,33,35,36,37,38,44,47,49].

For the primary efficacy measurement, DCR at 3 months after Ho-166-TARE, ten studies involving 180 patients were included in the analysis, with six studies using the RECIST 1.1 [30,32,35,38,39,47,49] and five using the mRECIST [30,32,33,36,37] response evaluation systems (Figure 2). One study, Braat [30], used both systems. However, in this study, not all patients could be evaluated according to mRECIST in addition to RECIST 1.1. Therefore, we included the RECIST 1.1 evaluation of this study in the overall DCR analysis.

RECIST 1.1 evaluates tumor response based on changes in the sum of the longest diameters of target lesions and defines complete response (CR) as the disappearance of all target lesions, partial response (PR) as a ≥30% decrease in the sum of the diameters of target lesions, progressive disease (PD) as a ≥20% increase in the sum (with an absolute increase of at least 5 mm), and stable disease (SD) as any response that does not meet criteria for PR or PD. In contrast, mRECIST accounts for treatment-induced necrosis by evaluating only the viable (contrast-enhancing) portion of the tumor on dynamic contrast-enhanced imaging in the arterial phase while applying the same percentage thresholds as RECIST 1.1. This distinction is particularly important in locoregional therapies like Ho-166-TARE, where tumor shrinkage may be minimal but necrosis is substantial.

The overall DCR was 72% (95% CI, 46–89%) (Figure 2). The DCR in the studies using RECIST 1.1 was 54% (95% CI, 22–83%), whereas the DCR in the studies that evaluated tumor response according to mRECIST was 93% (95% CI, 71–99%), indicating that Ho-166-TARE was especially effective in keeping the tumors from growing in the studies where tumor response could be evaluated by mRECIST (Figure 2). A detailed analysis of responders (complete response + partial response) across studies is provided in Supplementary Material S1.

In the RECIST 1.1 studies, between-study heterogeneity was severe, whereas in the mRECIST studies, the I2 test did not provide a reliable result, possibly due to the small number of studies included. However, we expect that heterogeneity would be similarly high in mRECIST studies as well (RECIST 1.1: I2 56–91%, *p* < 0.001; mRECIST: I2 0–79%, *p* = 0.516).

### 3.4. Survival

**Figure 3:**
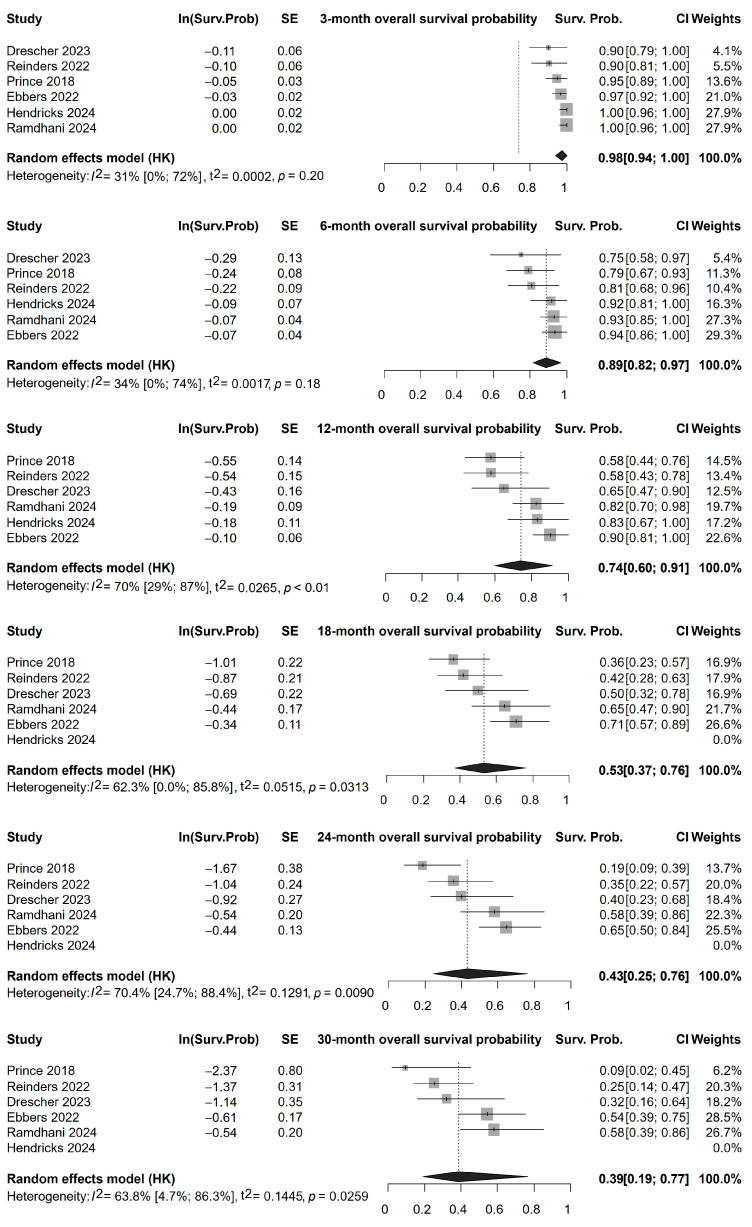
Overall survival rates of patients following Ho-166-TARE at different follow-up times until 30 months of follow-up [33,34,35,37,48,49].

The analysis of overall survival (OS) included six studies [33,34,35,37,48,49]. However, after 12 months, only four studies [33,34,35,37] could be included for analysis, with OS being assessed for up to 30 months. The pooled probability of overall survival was 98% (95% CI, 94–100%) at 3 months, 89% (95% CI, 82–97%) at 6 months, 74% (95% CI, 60–91%) at 12 months, 50% (95% CI, 31–81%) at 18 months, 39% (95% CI, 18–86%) at 24 months and 33% (95% CI, 13–83%) at 30 months (Figure 3).

The analysis of overall progression-free survival (PFS) included four studies [34,35,48,49]. The pooled probability of progression-free survival was 91% (95% CI, 68–100%) at 3 months, 69% (95% CI, 35–100%) at 6 months, 61% (95% CI, 24–100%) at 9 months, and 44% (95% CI, 5–100%) at 12 months (Supplementary Material S2). The forest plots and Kaplan–Meier curves (Figure 3, Supplementary Material S2) illustrate the survival outcomes with notable variation between studies.

### 3.5. Safety Assessment—Clinical and Laboratory Adverse Events

Clinical and laboratory adverse events were defined according to the CTCAE, the most commonly used standard for assessing the severity of adverse events. Adverse events of Grade 3 or higher were considered severe adverse events in every included study. To address variability in follow-up durations across studies, we standardized our adverse event analysis to include only adverse events reported up to 3 months, wherever the studies explicitly mentioned the time of adverse event assessment. This decision was made to minimize potential bias arising from differences in follow-up durations, as studies with extended observation periods may naturally report a higher incidence of adverse events over time. Additionally, focusing on the 3-month timeframe ensures a more reliable association between adverse events and Ho-166-TARE, as events occurring beyond this period may be influenced by disease progression or other treatment interventions rather than the direct effects of radioembolization.

Ten studies were included in the clinical safety analysis [30,32,33,35,36,37,38,39,48,49]. The proportion of severe clinical adverse events was close to zero in every case, indicating that Ho-166-TARE was safe. The individual forest plots can be seen in Supplementary Material S3. Radioembolization-induced liver disease (REILD)—manifested by jaundice, ascites, hyperbilirubinemia, and hypoalbuminemia in the absence of tumor progression or biliary obstruction—and radiation pneumonitis (RP)—a potential complication characterized by dry cough and exertional dyspnea—are serious complications of TARE. Only one confirmed REILD case was reported across the studies, and no RP cases were reported in any of the included articles [35,50,51,52].

**Figure 4:**
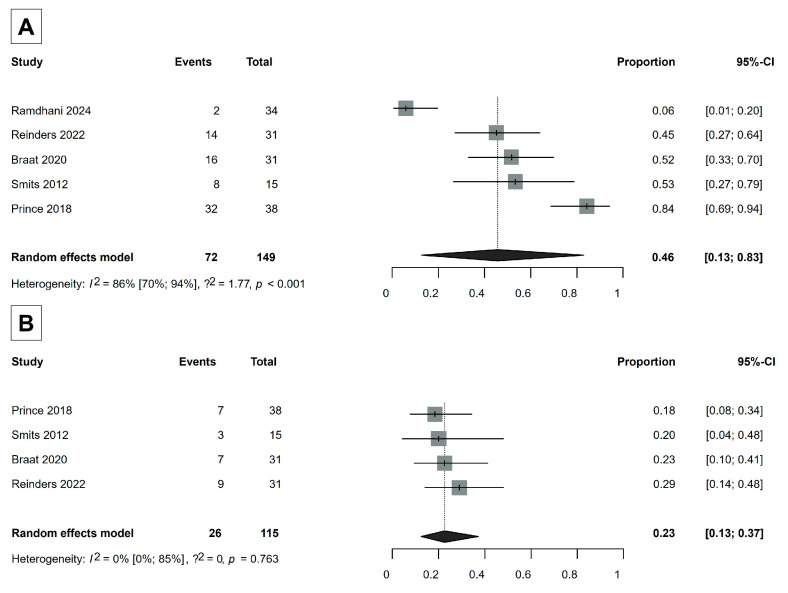
(**A**) Severe increase events in GGT levels (gamma-glutamyl transferase). (**B**) Development of severe lymphocytopenia [30,35,37,39,49].

Seven studies were included in the analysis of laboratory safety [30,32,35,36,37,39,49]. We observed severe changes in two laboratory parameters: an increase in gamma-glutamyl transferase (GGT) levels (>5 × upper limit normal if the baseline was normal; >5 × baseline if the baseline was abnormal) and the development of lymphocytopenia (<500 mm^3^). The proportion of severe GGT increase was 46% (95% CI, 13–83%), and the proportion of the development of severe lymphocytopenia was 23% (95% CI, 13–37%) (Figure 4). Otherwise, severe laboratory adverse events rarely occurred (Supplementary Material S3), indicating the safety of Ho-166-TARE.

### 3.6. Tumor-Absorbed Dose and Healthy Liver-Absorbed Dose

**Figure 5:**
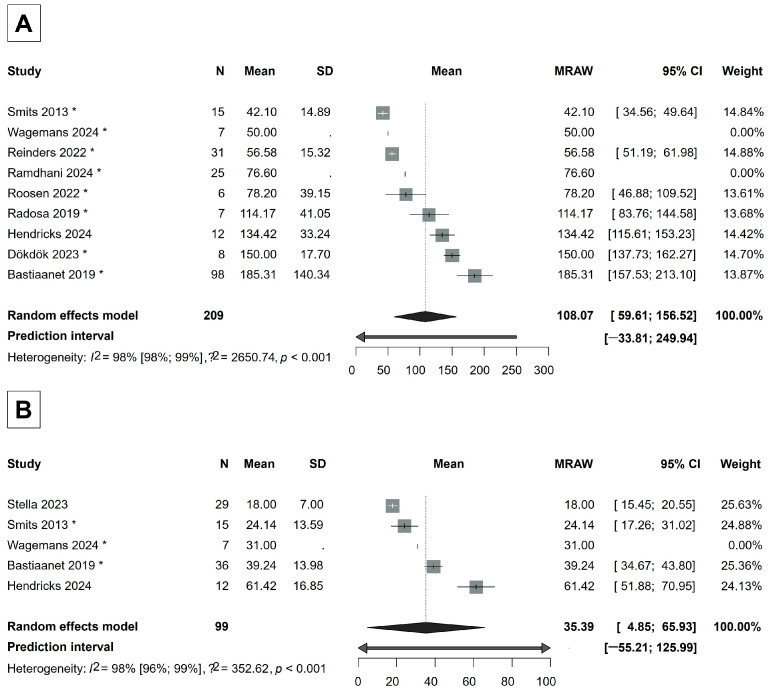
(**A**) Forest plot of the mean tumor-absorbed dose. (**B**) Forest plot of the mean healthy liver-absorbed dose. If a study is marked with *, then its mean and/or standard deviation is estimated from median, quartiles, or minimum, maximum values [31,32,36,37,38,44,45,47,48,49].

Dosimetry data were collected from 10 articles [31,32,36,37,38,44,45,47,48,49]. The mean tumor-absorbed dose was 108.07 Gy (95% CI, 59.61–156.52 Gy), almost three times higher than the dose absorbed by the mean healthy liver-absorbed dose, which was 35.39 Gy (95% CI, 4.85–65.93 Gy) (Figure 5).

### 3.7. Risk of Bias and Study Heterogeneity Assessment

We assessed study quality using the MINORS checklist [26]. The risk of bias was assessed separately for the analyses discussed above (Supplementary Material S4). The overall risk of bias for the studies ranged from low to moderate. In general, the most significant source of bias was due to inadequate study population size and selection bias, arising from the specific patient population referred for radioembolization therapy.

The studies included displayed considerable heterogeneity in study design, patient populations, and tumor types. This variability contributed to substantial between-study heterogeneity in the meta-analyses, as observed in outcomes such as DCR-s, overall survival (OS), and progression-free survival (PFS).

To further investigate publication bias and the potential sources of heterogeneity, we generated funnel plots and Baujat plots (Supplementary Material S7). The funnel plots helped assess small-study effects and reporting bias, while the Baujat plots identified individual studies contributing most to heterogeneity. These visual analyses supported the presence of substantial heterogeneity in certain outcomes and indicated that no single study disproportionately influenced the overall results.

## 4. Discussion

Transarterial radioembolization with Holmium-166 microspheres provides a targeted approach for the treatment of primary and secondary liver tumors when surgical options are limited. By delivering radiation directly to the tumor site, Ho-166-TARE aims to control tumor progression while minimizing the impact of radiation on healthy liver tissue. This study investigates the therapeutic potential of Ho-166-TARE by assessing both efficacy and safety.

### 4.1. Efficacy

The primary outcome of this study is the radiological response in terms of disease control rate (DCR: complete response, partial response, and stable disease according to RECIST 1.1 and mRECIST) at 3 months of follow-up after Ho-166-TARE [17,18,19,20]. Tumor response using RECIST 1.1 or mRECIST was reported in ten included studies [30,32,33,35,36,37,38,39,47,49] (Figure 2). The overall pooled DCR was 72%, the pooled DCR in the studies that used RECIST 1.1 was 54%, while in the studies that used mRECIST, the pooled DCR was 93%. The DCR in the studies that used mRECIST was explicitly higher than in the studies that used RECIST 1.1.

TARE delivers high doses of beta radiation, inducing DNA fragmentation and tumor necrosis [9,53]. The absorption of the necrotic mass leads to tumor shrinkage, although it may take several months to manifest, with a median response time of approximately 6 months after TARE [54,55]. In contrast, vascular enhancement changes can be measured earlier, around 2 months after TARE [54,55]. As TARE leads to a delayed volumetric response, RECIST 1.1, which primarily measures tumor shrinkage, may not always be suitable for accurately assessing tumor response. While RECIST 1.1 effectively reflects the antitumor activity of cytotoxic drugs, when applied to molecularly targeted therapies or locoregional treatments, anatomical tumor metrics may not reflect the early therapeutic effects reliably [20,56,57]. Due to the lack of long-term follow-up data, our analysis was based on a 3-month follow-up, which may explain the lower DCR (54%) observed in the studies that used RECIST 1.1. With longer follow-ups, tumor shrinkage could have become more noticeable over time [53,58].

In contrast, mRECIST was developed to assess viable tumor tissue based on arterial phase enhancement in contrast-enhanced imaging, thus providing an earlier and more precise evaluation of treatment-induced changes [20,56,57,59,60]. Although mRECIST is only validated in HCC patients, according to the EANM (European Association of Nuclear Medicine) procedure guideline, evaluating response using mRECIST criteria can be beneficial in hypervascular tumors, such as ICC or neuroendocrine neoplasms [8]. Our findings indicate that Ho-166-TARE effectively controlled tumor progression with a 3-month DCR of 93% (95% CI, 71–99%) by mRECIST, which was considerably higher than the DCR of 54% (95% CI, 22–83%) observed with RECIST 1.1. The reason could be that the studies that used mRECIST mostly treated HCC and other hypervascular lesions, which exhibit a better response to TARE, as their increased arterial supply allows higher microsphere accumulation (Figure 2) [61,62,63,64]. Another possible reason for this difference could be that mRECIST can provide an earlier evaluation of tumor response compared to RECIST 1.1 when it is applicable [65]. However, as tumor response following TARE typically continues to evolve beyond the 3-month timeframe, longer follow-up is necessary to fully assess the long-term therapeutic benefit.

Most of the articles included in our meta-analysis examined multiple tumor types. In the tumor response analysis, only Wagemans [47] (RECIST 1.1) and Radosa [36] (mRECIST) focused on a single tumor type: intrahepatic cholangiocarcinoma (ICC) [47] and hepatocellular carcinoma (HCC) [36], respectively. In line with our findings, both studies, which included hypervascular lesions, demonstrated good tumor response, with DCRs of 83% and 89% (Figure 2).

In a study by Smits [39], a particularly poor tumor response was observed (Figure 2). The underlying reason could be that this phase 1 study prioritized safety and dose optimization to protect healthy liver tissue rather than maximum tumor response, with lower initial Ho-166-TARE doses, which may have been less effective in destroying tumor cells.

Since mRECIST provides a more precise and earlier evaluation of tumor response compared to RECIST 1.1, it should be the preferred method for early post-TARE follow-up whenever it is applicable. Its ability to assess viable tumor response as early as three months offers a substantial advantage in monitoring treatment efficacy and making prompt clinical decisions.

Tumor-specific response assessment was not feasible with the available data, as most studies did not report tumor response separately for each tumor type. Additionally, most studies did not report the histologic subtypes of the included tumors, which further limited our ability to perform subtype-specific analyses. Future prospective studies should focus on evaluating tumor-specific responses to Ho-166-TARE and optimizing follow-up strategies across malignancies while also incorporating histopathological classification to enhance interpretability.

The considerable heterogeneity observed may be attributed to several factors. One contributor is the variability in tumor types, which differ in terms of vascularity, biology, and sensitivity to intra-arterial therapies. For instance, hypervascular tumors such as HCC are more likely to exhibit early imaging changes post TARE, particularly when evaluated with mRECIST, compared to hypovascular metastases. Additionally, tumor burden and extent can influence microsphere distribution and treatment efficacy, introducing further variability in response. Furthermore, patient selection factors such as age, sex, liver function, comorbidities, race/ethnicity, and previous treatments likely influence individual treatment response and tolerability. Similarly, variations in disease stage directly affect prognosis and can skew pooled survival and response rates. Lastly, differences in study design, including prospective versus retrospective methodology, single-center versus multicenter settings, and inconsistent follow-up durations, may lead to differences in data quality, completeness, and outcome definitions. Collectively, this clinical and methodological variability across studies likely underpins the observed inconsistencies in pooled estimates and complicates direct comparisons between studies.

### 4.2. Survival

Transarterial radioembolization with Ho-166-labeled microspheres appears to offer comparable overall and progression-free survival outcomes with other locoregional treatments [66,67]. Our overall and progression-free survival results (Figure 3, Supplementary Material S2) after Ho-166-TARE are consistent with those reported in meta-analyses evaluating the effects of Yttrium-90-TARE [14,68,69]. The survival outcomes reported in this meta-analysis were measured from the initiation of Ho-166-TARE. Therefore, our results may underestimate the effects of Ho-166-TARE on survival rates, given that some patients may have received prior systemic chemotherapy or other locoregional treatments (Supplementary Material S5).

However, assessing the long-term effectiveness of Ho-166-TARE in a heterogeneous patient population has limitations. The included studies featured a mix of primary and secondary liver malignancies at various disease stages, which may contribute to variations in survival outcomes. Additionally, differences in treatment history, baseline tumor burden (Supplementary Materials S5 and S6), and underlying liver function may further influence these results. Future studies with longer follow-up periods and tumor-specific subgroup analyses are important in determining the long-term efficacy of Ho-166-TARE in different patient populations.

Prince [35] reported a notably worse PFS than the other studies included in the analysis. This study focused on salvage patients with advanced disease stages. Additionally, the study population included a mix of primary tumor types with unique characteristics. These factors may have influenced poor PFS outcomes.

### 4.3. Safety

Up to three months after TARE, there were almost no severe (Grade 3 or higher according to CTCAE) clinical or laboratory adverse events (Supplementary Material S3). Our results indicate that Ho-166-TARE is safe, with minimal impact on critical liver functions, as only minor changes in liver function markers were detected.

The low incidence of side effects in Ho-166-TARE can be attributed to several factors. The treatment is highly selective, designed to deliver radiation directly to the tumor tissue while minimizing exposure to healthy liver parenchyma. Effective blood flow facilitates the accumulation of microspheres in the tumor’s capillary bed, thereby reducing the likelihood of non-target embolization and subsequent toxicity [58]. Due to the small size of the microspheres, Ho-166-TARE does not cause immediate ischemia and intense pain. Instead, it induces DNA strand breaks that inhibit tumor cell replication [9]. Although serious complications can occur if microspheres are delivered to sites unintended to be treated, such as the intestines or the gallbladder, this risk is minimized by a prior planning procedure to ensure precise targeting [70].

This targeted approach is reflected in the very low incidence of REILD, with only one confirmed case reported across the included studies. In addition, RP was not reported in any study, suggesting a low risk of pulmonary toxicity following Ho-166-TARE. A likely contributing factor to this absence of RP is the superior dosimetric accuracy of Ho-166-scout compared to the commonly used ^99m^Tc-MAA [50]. The scout dose capability of Ho-166 allows for a more precise prediction of absorbed lung radiation, enabling interventional radiologists to adjust the prescribed activity accordingly, minimizing lung toxicity [11,35,71].

It is important to note severe changes in two laboratory parameters: an increase in GGT levels and the development of lymphocytopenia (Figure 4). TARE can elevate GGT levels by causing localized liver cell damage, inflammation, and bile duct irritation, leading to cholestasis [72]. In addition, focused radiation in TARE significantly reduces lymphocyte counts through radiation-induced cell death, as lymphocytes, which are particularly susceptible to radiation, repeatedly pass through the tumor and are exposed to beta-radiation [73,74,75,76]. Our findings suggest that Ho-166-TARE may have a similar impact on liver function and immune response to Yttrium-90-TARE [75]. The modest increase in these markers shows that the treatment does not significantly disrupt liver function, supporting the safety profile of Ho-166-TARE.

Our study also highlights the importance of the tumor-absorbed dose in achieving treatment efficacy while maintaining safety. A higher tumor-absorbed dose can increase the likelihood of effectively killing tumor cells, but limiting the dose to healthy liver tissue is crucial for preventing REILD and other complications [42,52,77]. We found that the mean tumor-absorbed dose (108.07 Gy, 95% CI: 59.61–156.52 Gy) was almost three times higher than the mean dose absorbed by healthy liver tissue (35.39 Gy, 95% CI: 4.85–65.93 Gy) after Ho-166-TARE (Figure 5). While this confirms that Ho-166-TARE effectively spares healthy liver tissue, the tumor-absorbed dose in our analysis may not be sufficient to achieve optimal tumor response. A recent study by Reinders [78] investigated dose–response relationships in HCC patients treated with Ho-166-TARE and found that tumors achieving partial or complete response received 41% higher absorbed doses compared to non-responding tumors. The study identified a tumor-absorbed dose threshold of 155 Gy for a 90% probability of response and 184.5 Gy for a 100% response probability, suggesting that doses higher than those reported in our analysis may be necessary for maximal efficacy.

The majority of the studies included in our analysis were safety-focused trials, which often prioritized treatment tolerability over maximal tumor irradiation. Additionally, personalized dosimetry was not routinely applied in these studies, meaning treatment planning was based on standard dosing approaches rather than individualized tumor- and liver-specific dose calculations. From a safety perspective, our findings align with liver radiation tolerance limits, as the liver can typically withstand absorbed doses up to 60 Gy without failure [39,78]. The fact that the reported mean healthy-liver-absorbed dose in our analysis was 35.39 Gy further supports the favorable safety profile of Ho-166-TARE. However, as radioembolization moves towards more personalized dosimetry strategies, future studies should focus on optimizing tumor-absorbed doses while maintaining hepatic safety. These results reinforce the need for individualized dose planning, as recent data suggest that higher doses than those used in the included studies could lead to improved tumor response rates without compromising safety [78].

Given the heterogeneity in tumor types and extent across the studies, we included a summary of tumor types and sizes to support data interpretation and help guide future dose optimization efforts (Supplementary Material S6).

Although this meta-analysis includes data from studies that were conducted in various settings, a recent multicenter retrospective registry by Schulze-Zachau reinforces our conclusions [79]. Their real-world data confirmed similar safety and efficacy outcomes, highlighting that Ho-166 TARE can achieve outcomes consistent with recent controlled studies [35,37]. The multicenter design of this study strengthens the external validity of our findings and reinforces the applicability of the therapy in different clinical settings. This alignment between controlled and real-world data highlights the robustness of Holmium-166 TARE as a treatment modality for primary and secondary liver malignancies [80].

### 4.4. Strengths and Limitations

This systematic review and meta-analysis is the first comprehensive evaluation of the safety and efficacy of Ho-166-TARE in treating primary or secondary liver tumors. We provide an overview of the clinical outcomes associated with this innovative therapy by including data from multiple interventional and observational studies.

This meta-analysis follows strict guidelines and uses a methodologically sound approach to answer the research question.

The inclusion of DCR based on both RECIST 1.1 and mRECIST adds valuable insight, highlighting the differences in tumor response evaluation between anatomical and functional imaging methods.

Heterogeneity across the included studies is a major limitation, as variability in study designs, patient populations, prior treatments, tumor types, tumor stages, and treatment protocols may have influenced the pooled results, particularly in terms of progression-free survival (PFS) and overall survival (OS). Additionally, most studies reported aggregated data on primary and secondary liver tumors. This may have contributed to clinical heterogeneity and limits tumor-specific interpretation of treatment outcomes.

Another limitation is the short follow-up duration in most studies. Among the studies included in the response analysis, only two studies by Braat [30] and Prince [35] reported tumor response at 6 months of follow-up, and only Prince [33] tracked tumor response for up to 9 months. Therefore, a comprehensive analysis at longer follow-up times was not possible. This limits the ability to draw conclusions about sustained tumor control, survival, or long-term adverse events over extended periods. Further data with increased follow-up periods are needed to better understand the long-term efficacy of Ho-166-TARE.

Additionally, although mRECIST is officially validated only for HCC, it was also used to assess response in other hypervascular tumors. While this approach aligns with EANM guidelines, suggesting its applicability in ICC and neuroendocrine neoplasms, the lack of formal validation for non-HCC tumors may introduce variability in response assessment [8]. Further research is required to standardize response criteria for Ho-166-TARE in non-HCC malignancies.

Lastly, most included studies use standard dosimetry rather than personalized dosimetry, which may limit the generalizability of our results [81]. Recent data suggest that higher tumor-absorbed doses could lead to improved response rates, reinforcing the need for individualized dosimetry protocols in future studies [78].

### 4.5. Implications for Practice and Research

In clinical practice, patient selection based on tumor characteristics is important to achieve better outcomes. Our findings suggest that hypervascular tumors demonstrate particularly favorable responses, reinforcing the value of mRECIST for response evaluation whenever applicable. This study supports Ho-166-TARE as a safe and effective option for treating liver tumors, with a favorable safety profile and results comparable to Yttrium-90-TARE (Supplementary Materials S1 and S3) [82].

Future research should focus on longer follow-up durations to fully assess the long-term efficacy and safety of Ho-166-TARE. Additionally, most of the included studies did not stratify responses by tumor type, limiting the ability to draw tumor-specific conclusions. Future research should aim to fill this gap by conducting subgroup analyses to determine how different malignancies respond to Ho-166-TARE. Another critical aspect to consider is the refinement of personalized dosimetry. While Ho-166-TARE has demonstrated a favorable safety profile, recent evidence suggests that higher tumor-absorbed doses could enhance response rates without compromising liver function. Therefore, optimizing dose delivery strategies is essential to maximize therapeutic efficacy while maintaining hepatic safety.

This study was conducted as part of the translational medicine framework proposed by the Academia Europaea. Accordingly, it aimed to bridge the gap between clinical research and medical practice [83,84].

## 5. Conclusions

Transarterial Radioembolization with Ho-166-labeled microspheres presents a safe and effective treatment option for patients with primary or secondary liver tumors. Our findings suggest that mRECIST is more suitable for evaluating early treatment response in hypervascular tumors, which tend to respond better to Ho-166-TARE. This highlights the need for tumor-specific response assessment strategies to ensure the most accurate evaluation of treatment efficacy. This study emphasizes the need for longer follow-up periods to assess long-term efficacy and safety. In addition, future research should explore the optimization of tumor-absorbed doses and the role of personalized dosimetry. Larger, prospective clinical trials with extended follow-up periods are essential to establish standardized protocols and further define the role of Ho-166-TARE in liver-directed therapy.

## Figures and Tables

**Table 1:** Summary of the included articles.

First Author, Publication Date	Country, Centers	Study Period	Study Design	Number of Patients	Age, Years (Mean (SD) or Median (Range))	Sex, Female % of Total	Primary Tumor	Tumor Response Evaluation Criteria
Braat et al., 2020 [30]	The Netherlands, single center	October 2014–September 2018	Prospective, nonrandomized, noncomparative clinical trial	30	62 (8)	27	Pancreas, ileum/jejunum, CRC, bronchus/lung, unknown	RECIST 1.1 mRECIST
Bastiaannet et al., 2019 [31]	The Netherlands, single center	2009–2015	Prospective, nonrandomized, noncomparative clinical trial	36	64 (40–84)	53	Breast, ICC, melanoma, neuroendocrine neoplasm, thymus, pancreas, CRC	Not included in the response analysis
Dökdök et al., 2023 [32]	Turkey, single center	January 2019–February 2020	Retrospective case cohort	9	56 (12)	78	Breast, melanoma, pancreas, CRC, gastric, ovary	RECIST 1.1 mRECIST
Drescher et al., 2023 [33]	Germany, single center	February 2019–March 2021	Prospective cohort	20	69.5 (57–82)	25	HCC, ICC, CRC, liver haemangioendothelioma,	mRECIST
Ebbers et al., 2022 [34]	The Netherlands, single center	Not reported	Prospective cohort	31	65.1 (IQR: 57.6–70.2)	26	Pancreas, ileum/jejunum, CRC, bronchus/lung, unknown	Not included in the response analysis
Prince et al., 2018 [35]	The Netherlands, single center	May 2012–March 2015	Prospective, nonrandomized, noncomparative clinical trial	38	66 (41–84)	42	Breast, ICC, melanoma, neuroendocrine neoplasm, thymus, pancreas, CRC, gastric	RECIST 1.1
Radosa et al., 2019 [36]	Germany, single center	March 2017–April 2018	Retrospective cohort	9	73 (64–78)	11	HCC	mRECIST
Reinders et al., 2022 [37]	Multicentre	December 2017–January 2020	Prospective nonrandomized, noncomparative clinical trial	31	73 (44–85)	10	Unknown	mRECIST
Roosen et al., 2022 [38]	The Netherlands, single center	Not reported	Prospective nonrandomized, noncomparative clinical trial	6	67 (32–81)	67	HCC, breast, ICC, CRC	RECIST 1.1
Smits et al., 2012 [39]	The Netherlands, single center	November 2009–September 2011	Prospective nonrandomized, noncomparative clinical trial	15	55 (38–87)	40	Breast, ICC, melanoma, CRC	RECIST 1.1
Roekel et al., 2021 [42]	The Netherlands, single center	Not reported	Retrospective cohort	40	64 (37–84)	37.5	CRC	RECIST 1.1
Smits et al., 2013 [44]	The Netherlands, single center	Not reported	Prospective nonrandomized, noncomparative clinical trial	15	56 (38–87)	40	Breast, ICC, melanoma, CRC	Not included in the response analysis
Stella et al., 2023 [45]	The Netherlands, single center	Not reported	Prospective nonrandomized, noncomparative clinical trial	31	65.1 (IQR: 57.6–70.2)	26	Pancreas, ileum/jejunum, CRC, bronchus/lung, unknown	Not included in the response analysis
Wagemans et al., 2024 [47]	The Netherlands, single center	June 2011–March 2020	Retrospective cohort	7	59 (45–83)	71	ICC	RECIST 1.1
Hendricks et al., 2024 [48]	Multicentre	April 2018–March 2021	Prospective nonrandomized, noncomparative clinical trial	12	66.5 (IQR: 64.3–71.7)	17	HCC	Not included in the response analysis
Randhani et al., 2024 [49]	The Netherlands, single center	2012–2022	Retrospective cohort	29	60 (mean) (25–80)	45	Neuroendocrine neoplasm	RECIST 1.1

ICC = intrahepatic cholangiocarcinoma. CRC = colorectal cancer. HCC = hepatocellular carcinoma. RECIST 1.1 = Response Evaluation Criteria in Solid Tumors 1.1. mRECIST = Modified Response Evaluation Criteria in Solid Tumor.

## Data Availability

The datasets generated during and/or analyzed in the current study are available from the corresponding author upon reasonable request.

## Supplementary Materials

The following supporting information can be downloaded at: https://www.mdpi.com/article/10.3390/cancers17111841/s1, Supplementary Material S1: Responders, Supplementary Material S2: PFS and Kaplan-Meier plots, Supplementary Material S3: Severe adverse events, Supplementary Material S4: Risk of bias assessment, Supplementary Material S5: Prior and concurrent therapies, Supplementary Material S6: Tumor extent and primary tumor type, Supplementary Material S7: Funnel plots and Baujat plots, Supplementary Material S8: Literature string.

## Abbreviations

The following abbreviations are used in this manuscript:

ICC	Intrahepatic cholangiocarcinoma
CR	Complete response
CRC	Colorectal cancer
CTCAE	Common terminology criteria for adverse events
DCR	Disease control rate
GGT	Gamma-glutamyl transferase
HCC	Hepatocellular carcinoma
Ho-166-TARE	Transarterial radioembolization using Holmium-166 microspheres
MINORS	Methodological index for non-randomized studies
mRECIST	Modified response evaluation criteria in solid tumors
OS	Overall survival
PD	Progressive disease
PFS	Progression-free survival
PR	Partial response
RECIST 1.1	Response evaluation criteria in solid tumors version 1.1
REILD	Radioembolization-induced liver disease
RP	Radiation pneumonitis
SD	Stable disease
TACE	Transarterial chemoembolization
TAE	Transarterial embolization
TARE	Transarterial radioembolization
