# Supplementary material for: Holmium-166 Radioembolization Is a Safe and Effective Locoregional Treatment for Primary and Secondary Liver Tumors: A Systematic Review and Meta-Analysis
Source: Cancers (Basel). 2025 May 31;17(11):1841. doi: 10.3390/cancers17111841 (PMC12153601; doi:10.3390/cancers17111841)

Progression-free survival rates in patients after Ho-166-TARE treatment at different follow-up times:

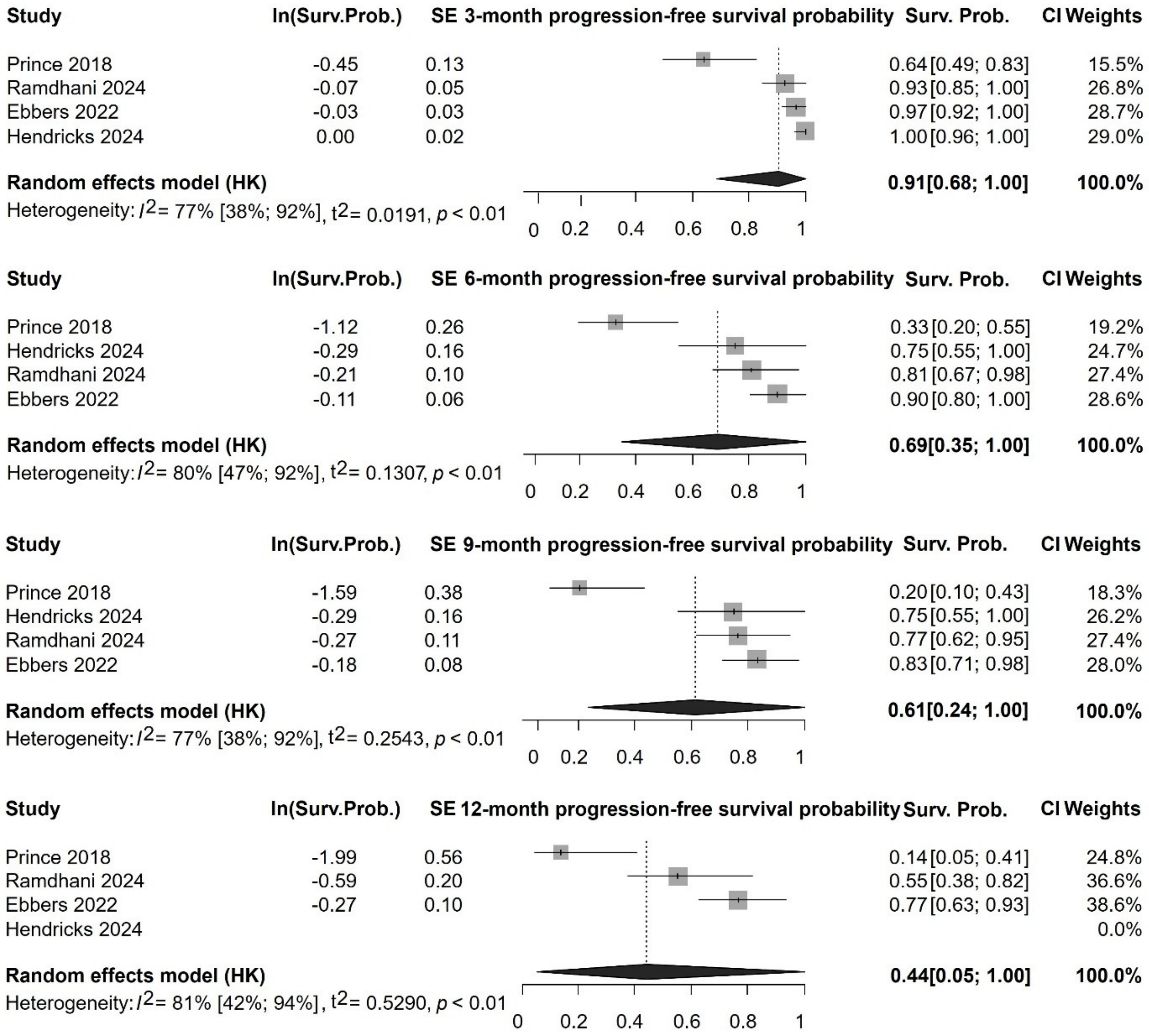

Kaplan-Meier plots. A: overall survival; B: progression-free survival:

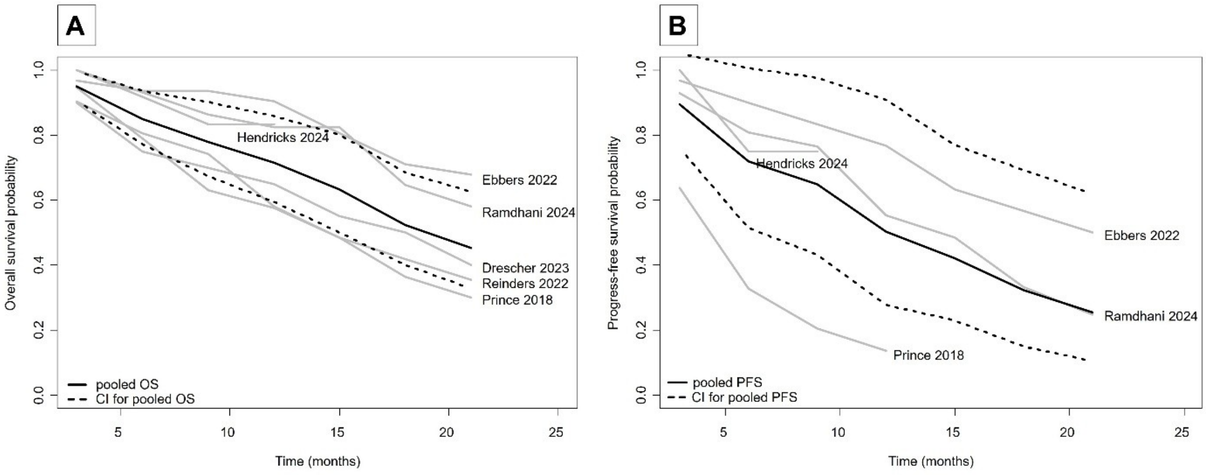

Supplement: Supplementary file 1 [file cancers-17-01841-s001.zip › Supplementary material 2_PFS and Kaplan-Meier plots.pdf]
