# Supplementary material for: Holmium-166 Radioembolization Is a Safe and Effective Locoregional Treatment for Primary and Secondary Liver Tumors: A Systematic Review and Meta-Analysis
Source: Cancers (Basel). 2025 May 31;17(11):1841. doi: 10.3390/cancers17111841 (PMC12153601; doi:10.3390/cancers17111841)

Forest plots of severe (grade 3 or higher) clinical adverse events according to Common Terminology Criteria for Adverse Events (CTCAE):

Severe (grade 3 or higher) abdominal infection:

Study	Events	Total	Proportion	95%-CI
Ramdhani 2024	1	34	0.03	[0.00; 0.15]
Reinders 2022	1	31	0.03	[0.00; 0.17]
Hendricks 2024	1	12	0.08	[0.00; 0.38]
**Random effects model**	**3**	**77**	**0.04**	**[0.00; 0.34]**

Heterogeneity: I^2 = 0% [0%; 90%] , ?^{2} = 0 , p = 0.708

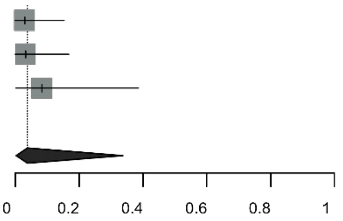

Severe (grade 3 or higher) abdominal pain:

Study	Events	Total	Proportion	95%-CI
Ramdhani 2024	0	34	0.00	[0.00; 0.10]
Drescher 2023	0	20	0.00	[0.00; 0.17]
Reinders 2022	1	31	0.03	[0.00; 0.17]
Radosa 2019	0	9	0.00	[0.00; 0.34]
Smits 2012	1	15	0.07	[0.00; 0.32]
Braat 2020	3	31	0.10	[0.02; 0.26]
Roosen 2022	1	6	0.17	[0.00; 0.64]
Prince 2018	7	38	0.18	[0.08; 0.34]
Dökdök 2023	2	9	0.22	[0.03; 0.60]
**Random effects model**	**15**	**193**	**0.06**	**[0.02; 0.16]**

Heterogeneity: I^2 = 0% [0%; 65%], ?^{2} = 0.68, p = 0.793

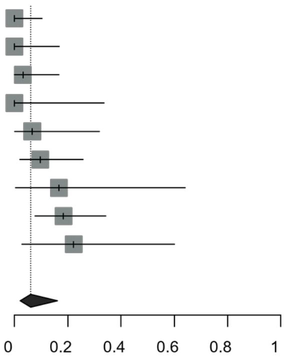

Severe (grade 3 or higher) allergic reaction:

Study	Events	Total	Proportion	95%-CI
Prince 2018	0	38	0.00	[0.00; 0.09]
Smits 2012	0	15	0.00	[0.00; 0.22]
Reinders 2022	1	31	0.03	[0.00; 0.17]
**Random effects model**	**1**	**84**	**0.01**	**[0.00; 0.48]**

Heterogeneity: I^2 = 0% [0%; 90%] , ?^{2} = 0 , p = 1.000

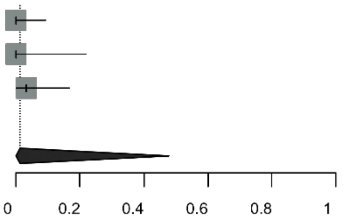

Severe (grade 3 or higher) ascites:

Study	Events	Total	Proportion	95%-CI
Prince 2018	1	38	0.03	[0.00; 0.14]
Smits 2012	0	15	0.00	[0.00; 0.22]
Reinders 2022	1	31	0.03	[0.00; 0.17]
Radosa 2019	0	9	0.00	[0.00; 0.34]
Dökdök 2023	1	9	0.11	[0.00; 0.48]
**Random effects model**	**3**	**102**	**0.03**	**[0.01; 0.13]**

Heterogeneity: I^2 = 0% [0%; 79%] , ?^{2} = 0 , p = 0.868

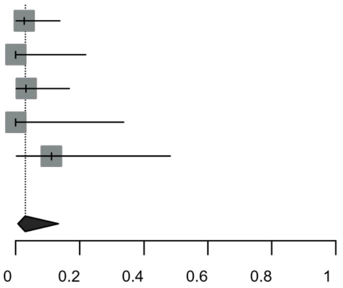

Severe (grade 3 or higher) back pain:

Study	Events	Total	Proportion	95%-CI
Prince 2018	0	38	0.00	[0.00; 0.09]
Ramdhani 2024	0	34	0.00	[0.00; 0.10]
Braat 2020	0	31	0.00	[0.00; 0.11]
Reinders 2022	0	31	0.00	[0.00; 0.11]
Roosen 2022	0	6	0.00	[0.00; 0.46]
**Random effects model**	**0**	**140**	**0.00**	**[0.00; 1.00]**

Heterogeneity: I^2 = 0% [0%; 79%] , ?^{2} = 0 , p = 1.000

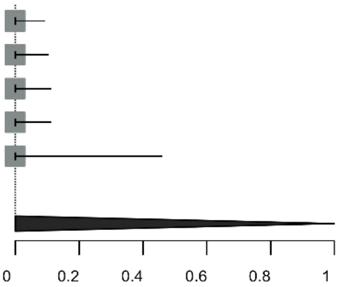

Severe (grade 3 or higher) constipation:

Study	Events	Total	Proportion	95%-CI
Prince 2018	0	38	0.00	[0.00; 0.09]
Braat 2020	0	31	0.00	[0.00; 0.11]
Smits 2012	0	15	0.00	[0.00; 0.22]
**Random effects model**	**0**	**84**	**0.00**	**[0.00; 1.00]**

Heterogeneity: I^2 = 0% [0%; 90%] , ?^{2} = 0 , p = 1.000

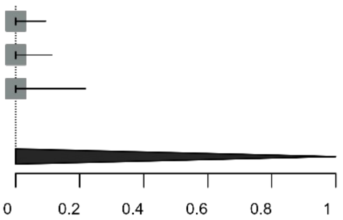

Severe (grade 3 or higher) dizziness:

Study	Events	Total	Proportion	95%-CI
Prince 2018	0	38	0.00	[0.00; 0.09]
Ramdhani 2024	0	34	0.00	[0.00; 0.10]
Reinders 2022	0	31	0.00	[0.00; 0.11]
Smits 2012	0	15	0.00	[0.00; 0.22]
**Random effects model**	**0**	**118**	**0.00**	**[0.00; 1.00]**

Heterogeneity: I^2 = 0% [0%; 85%] , ?^{2} = 0 , p = 1.000

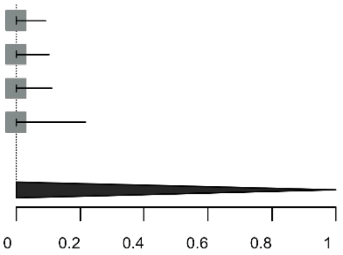

Severe (grade 3 or higher) dyspnea:

Study	Events	Total	Proportion	95%-CI
Prince 2018	0	38	0.00	[0.00; 0.09]
Ramdhani 2024	0	34	0.00	[0.00; 0.10]
Braat 2020	0	31	0.00	[0.00; 0.11]
Reinders 2022	0	31	0.00	[0.00; 0.11]
**Random effects model**	**0**	**134**	**0.00**	**[0.00; 1.00]**

Heterogeneity: I^2 = 0% [0%; 85%], ?^{2} = 0, p = 1.000

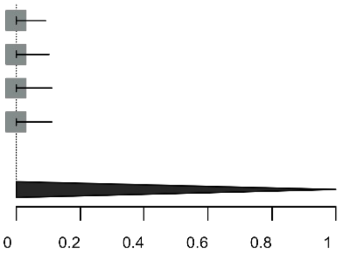

Severe (grade 3 or higher) fatigue:

Study	Events	Total	Proportion	95%-CI
Ramdhani 2024	0	34	0.00	[0.00; 0.10]
Reinders 2022	0	31	0.00	[0.00; 0.11]
Prince 2018	1	38	0.03	[0.00; 0.14]
Smits 2012	0	15	0.00	[0.00; 0.22]
Braat 2020	1	31	0.03	[0.00; 0.17]
Hendricks 2024	0	12	0.00	[0.00; 0.26]
Dökdök 2023	0	9	0.00	[0.00; 0.34]
Radosa 2019	0	9	0.00	[0.00; 0.34]
Roosen 2022	0	6	0.00	[0.00; 0.46]
**Random effects model**	**2**	**185**	**0.01**	**[0.00; 0.05]**

Heterogeneity: I^2 = 0% [0%; 65%], ?^{2} = 0, p = 1.000

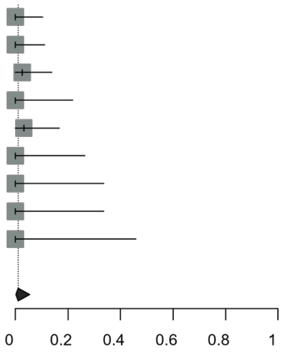

Severe (grade 3 or higher) fever:

Study	Events	Total	Proportion	95%-CI
Prince 2018	0	38	0.00	[0.00; 0.09]
Braat 2020	0	31	0.00	[0.00; 0.11]
Reinders 2022	0	31	0.00	[0.00; 0.11]
Dökdök 2023	0	9	0.00	[0.00; 0.34]
Radosa 2019	0	9	0.00	[0.00; 0.34]
Roosen 2022	0	6	0.00	[0.00; 0.46]
**Random effects model**	**0**	**124**	**0.00**	**[0.00; 1.00]**

Heterogeneity: I^2 = 0% [0%; 75%], ?^{2} = 0, p = 1.000

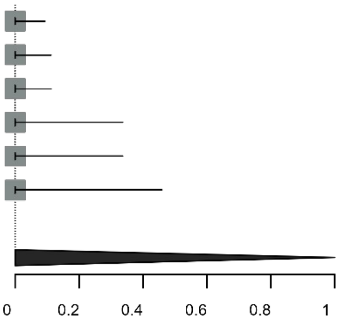

Severe (grade 3 or higher) gastrointestinal bleeding:

Study	Events	Total	Proportion	95%-CI
Prince 2018	1	38	0.03	[0.00; 0.14]
Braat 2020	1	31	0.03	[0.00; 0.17]
Reinders 2022	3	31	0.10	[0.02; 0.26]
**Random effects model**	**5**	**100**	**0.05**	**[0.01; 0.27]**

Heterogeneity: I^2 = 0% [0%; 90%] , ?^{2} = 0 , p = 0.396

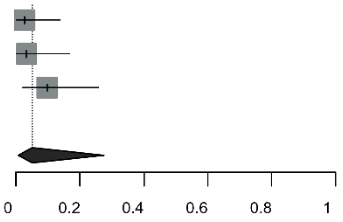

Severe (grade 3 or higher) joint pain:

Study	Events	Total	Proportion	95%-CI
Prince 2018	0	38	0.00	[0.00; 0.09]
Braat 2020	0	31	0.00	[0.00; 0.11]
Reinders 2022	1	31	0.03	[0.00; 0.17]
**Random effects model**	**1**	**100**	**0.01**	**[0.00; 0.43]**

Heterogeneity: I^2 = 0% [0%; 90%] , ?^{2} = 0 , p = 1.000

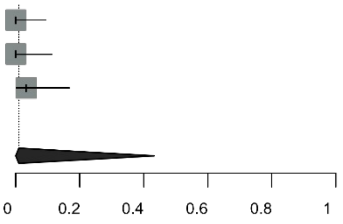

Severe (grade 3 or higher) nausea:

Study	Events	Total	Proportion	95%-CI
Reinders 2022	0	31	0.00	[0.00; 0.11]
Roekel 2021	0	21	0.00	[0.00; 0.16]
Smits 2012	0	15	0.00	[0.00; 0.22]
Braat 2020	1	31	0.03	[0.00; 0.17]
Radosa 2019	0	9	0.00	[0.00; 0.34]
Roosen 2022	0	6	0.00	[0.00; 0.46]
Prince 2018	3	38	0.08	[0.02; 0.21]
Dökdök 2023	2	9	0.22	[0.03; 0.60]
**Random effects model**	**6**	**160**	**0.03**	**[0.00; 0.16]**

Heterogeneity: I^2 = 0% [0%; 68%] , ?^{2} = 0.86 , p = 0.890

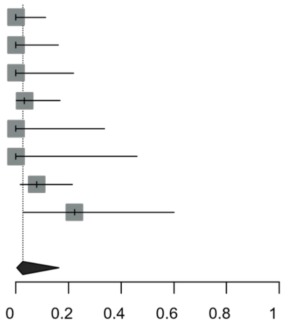

Severe (grade 3 or higher) oedema:

Study	Events	Total	Proportion	95%-CI
Prince 2018	0	38	0.00	[0.00; 0.09]
Braat 2020	0	31	0.00	[0.00; 0.11]
Reinders 2022	0	31	0.00	[0.00; 0.11]
Smits 2012	3	15	0.20	[0.04; 0.48]
**Random effects model**	**3**	**115**	**0.00**	**[0.00; 0.98]**

Heterogeneity: I^2 = 0% [0%; 85%] , ?^{2} = 9.62 , p = 1.000

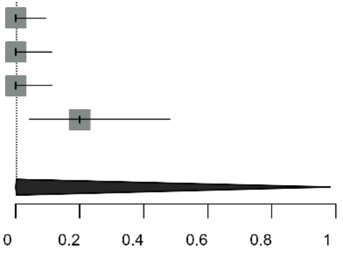

Severe (grade 3 or higher) radioembolization induced liver diesease:

Study	Events	Total	Proportion	95%-CI
Ramdhani 2024	0	34	0.00	[0.00; 0.10]
Prince 2018	1	38	0.03	[0.00; 0.14]
Braat 2020	1	31	0.03	[0.00; 0.17]
Dökdök 2023	0	9	0.00	[0.00; 0.34]
Radosa 2019	0	9	0.00	[0.00; 0.34]
**Random effects model**	**2**	**121**	**0.02**	**[0.00; 0.11]**

Heterogeneity: I^2 = 0% [0%; 79%], ?^{2} = 0, p = 1.000

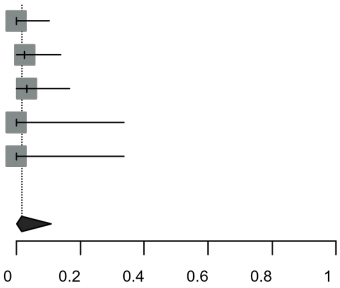

Severe (grade 3 or higher) vomiting:

Study	Events	Total	Proportion	95%-CI
Braat 2020	0	31	0.00	[0.00; 0.11]
Prince 2018	1	38	0.03	[0.00; 0.14]
Smits 2012	0	15	0.00	[0.00; 0.22]
Ramdhani 2024	0	12	0.00	[0.00; 0.26]
Radosa 2019	0	9	0.00	[0.00; 0.34]
**Random effects model**	**1**	**105**	**0.01**	**[0.00; 0.14]**

Heterogeneity: I^2 = 0% [0%; 79%], ?^{2} = 0, p = 1.000

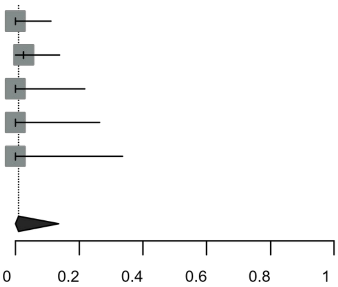

Forest plots of severe (grade 3 or higher) laboratory adverse events according to Common Terminology Criteria for Adverse Events (CTCAE):

Severe (grade 3 or higher) albumin decrease:

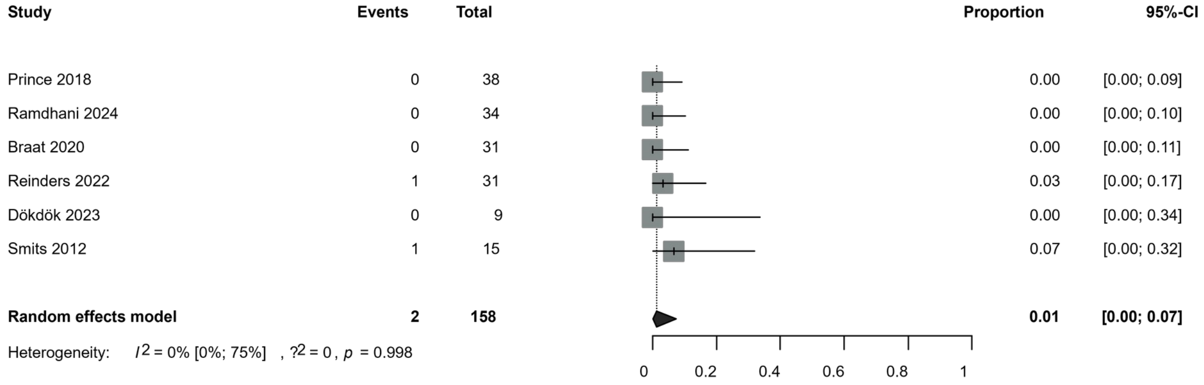

Severe (grade 3 or higher) ALKP increase:

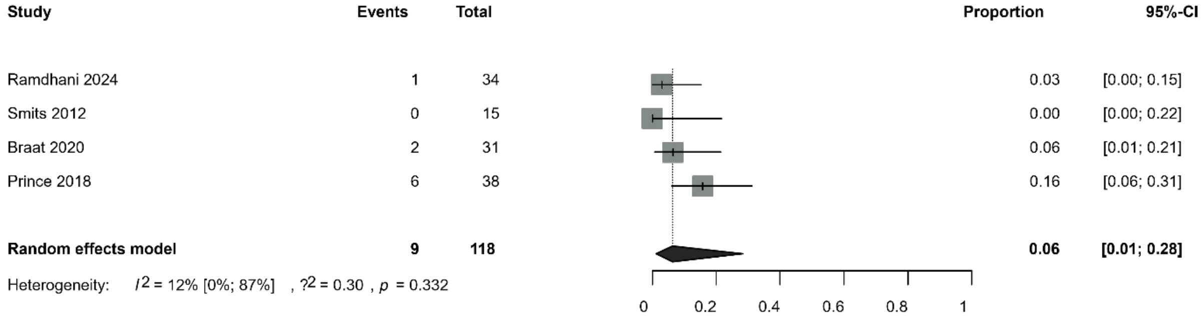

Severe (grade 3 or higher) ALT increase:

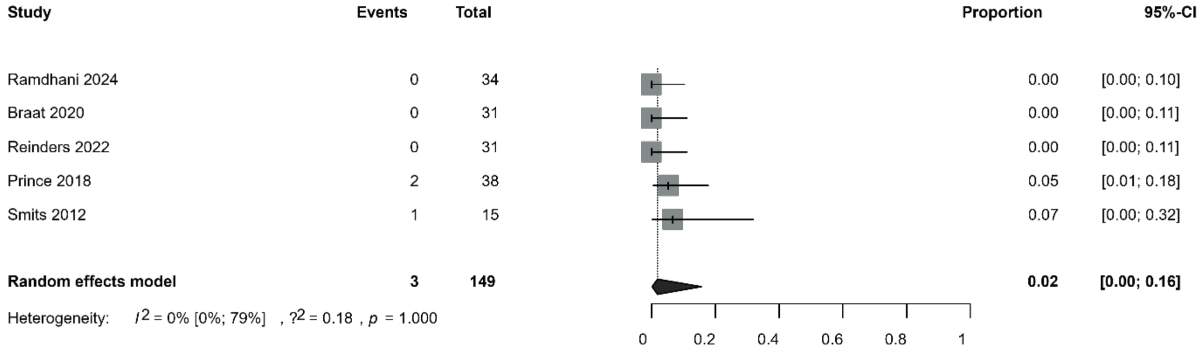

Severe (grade 3 or higher) anaemia:

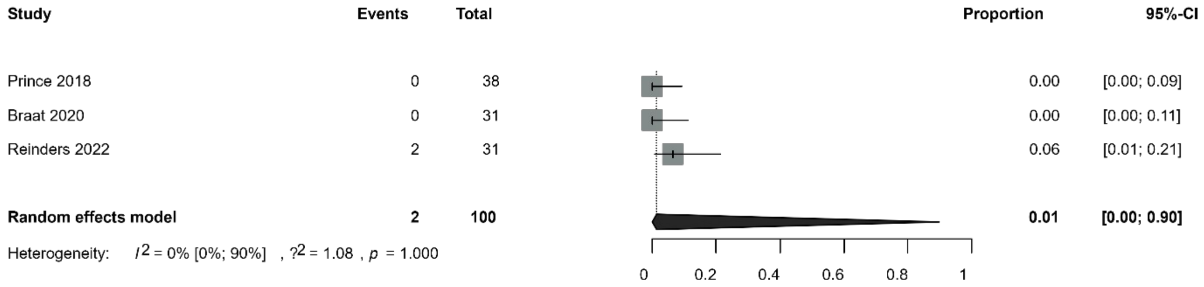

Severe (grade 3 or higher) AST increase:

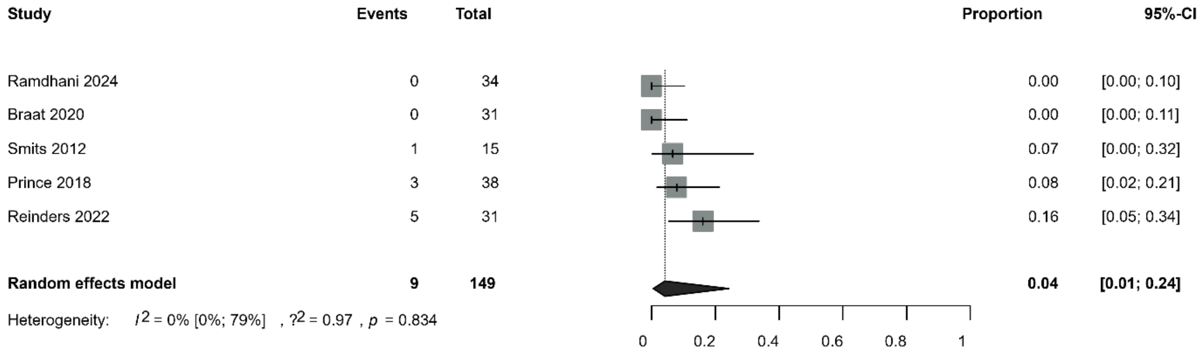

Severe (grade 3 or higher) creatinine increase:

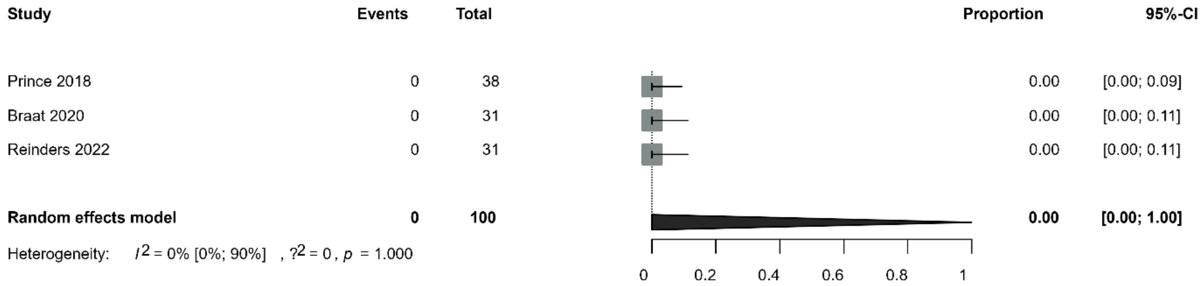

Severe (grade 3 or higher) GGT increase:

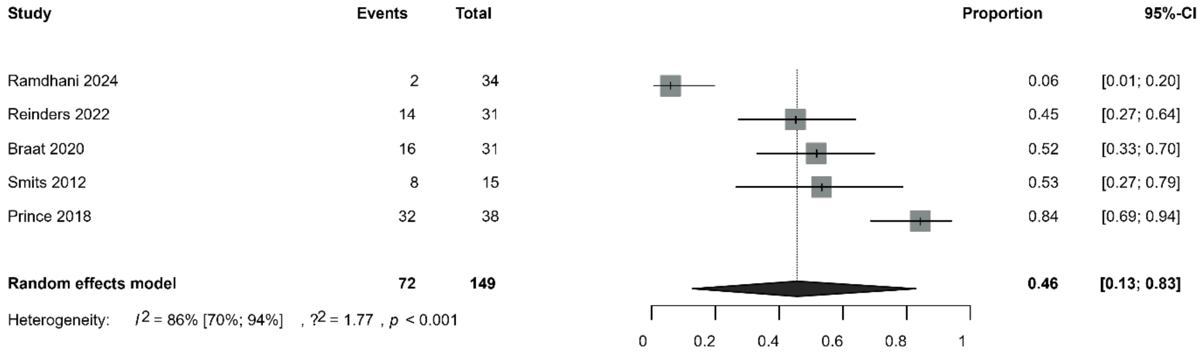

Severe (grade 3 or higher) LDH increase:

Study	Events	Total	Proportion	95%-CI
Prince 2018	0	38	0.00	[0.00; 0.09]
Ramdhani 2024	0	34	0.00	[0.00; 0.10]
Braat 2020	0	31	0.00	[0.00; 0.11]
Smits 2012	2	15	0.13	[0.02; 0.40]
**Random effects model**	**2**	**118**	**0.00**	**[0.00; 0.96]**

Heterogeneity: I^2 = 0% [0%; 85%] , ?^{2} = 6.53 , p = 1.000

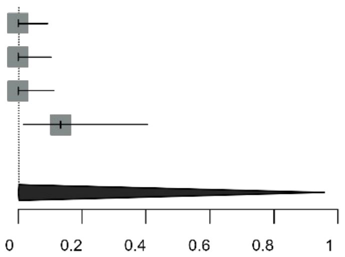

Severe (grade 3 or higher) leucopenia:

Study	Events	Total	Proportion	95%-CI
Prince 2018	0	38	0.00	[0.00; 0.09]
Braat 2020	0	31	0.00	[0.00; 0.11]
Smits 2012	1	15	0.07	[0.00; 0.32]
**Random effects model**	**1**	**84**	**0.01**	**[0.00; 0.91]**

Heterogeneity: I^2 = 0% [0%; 90%] , ?^{2} = 0.28 , p = 1.000

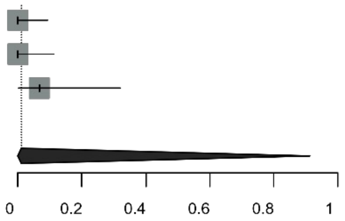

Severe (grade 3 or higher) lymphocytopenia:

Study	Events	Total	Proportion	95%-CI
Prince 2018	7	38	0.18	[0.08; 0.34]
Smits 2012	3	15	0.20	[0.04; 0.48]
Braat 2020	7	31	0.23	[0.10; 0.41]
Reinders 2022	9	31	0.29	[0.14; 0.48]
**Random effects model**	**26**	**115**	**0.23**	**[0.13; 0.37]**

Heterogeneity: I^2 = 0% [0%; 85%] , ?^{2} = 0 , p = 0.763

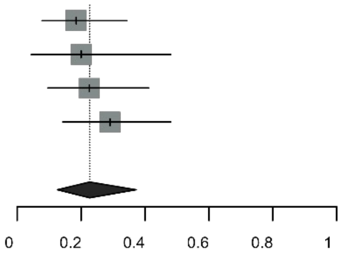

Severe (grade 3 or higher) thrombocytopenia:

Study	Events	Total	Proportion	95%-CI
Prince 2018	0	38	0.00	[0.00; 0.09]
Braat 2020	0	31	0.00	[0.00; 0.11]
Reinders 2022	0	31	0.00	[0.00; 0.11]
Smits 2012	1	15	0.07	[0.00; 0.32]
**Random effects model**	**1**	**115**	**0.01**	**[0.00; 0.84]**

Heterogeneity: I^2 = 0% [0%; 85%] , ?^{2} = 0.37 , p = 1.000

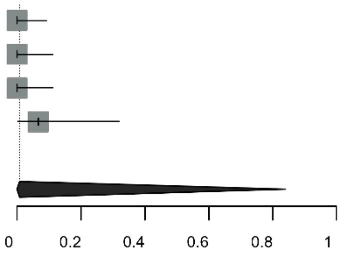

Severe (grade 3 or higher) total bilirubin increase:

Study	Events	Total	Proportion	95%-CI
Ramdhani 2024	0	34	0.00	[0.00; 0.10]
Prince 2018	1	38	0.03	[0.00; 0.14]
Braat 2020	1	31	0.03	[0.00; 0.17]
Reinders 2022	1	31	0.03	[0.00; 0.17]
Dökdök 2023	0	9	0.00	[0.00; 0.34]
Radosa 2019	0	9	0.00	[0.00; 0.34]
Smits 2012	1	15	0.07	[0.00; 0.32]
**Random effects model**	**4**	**167**	**0.02**	**[0.01; 0.08]**

Heterogeneity: I^2 = 0% [0%; 71%] , ?^{2} = 0 , p = 0.998

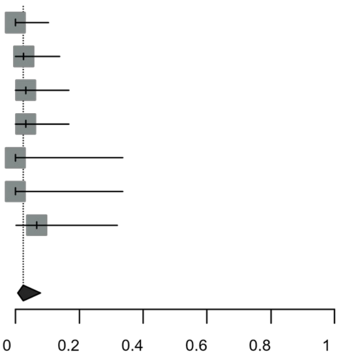

Supplement: Supplementary file 1 [file cancers-17-01841-s001.zip › Supplementary material 3_Severe adverse events.pdf]
