# Supplementary material for: Holmium-166 Radioembolization Is a Safe and Effective Locoregional Treatment for Primary and Secondary Liver Tumors: A Systematic Review and Meta-Analysis
Source: Cancers (Basel). 2025 May 31;17(11):1841. doi: 10.3390/cancers17111841 (PMC12153601; doi:10.3390/cancers17111841)

<u>Risk of bias assessment according to the methodological index for non-randomized studies (MINORS) per outcome: </u>

Disease control rate (DCR) according to RECIST 1.1 at 3 months follow-up:

Risk of bias

Study	D1	D2	D3	D4	D5	D6	D7	D8	Overall
Smits 2012	+	X	+	+	+	+	-	X	-
Roosen 2022	+	X	+	+	X	+	+	X	-
Prince 2018	+	X	+	+	+	+	+	+	+
Ramdhani 2024	+	-	-	+	X	+	-	X	-
Wagemans 2018	+	X	+	+	X	+	-	X	-
Braat 2020	+	X	+	+	+	-	+	+	+

D1: A clearly stated aim
D2: Inclusion of consecutive patients
D3: Prospective collection of data
D4: Endpoints appropriate to the aim of the study
D5: Unbiased assessment of the sudy endpoint
D6: Follow-up period appropriate to the aim of the study
D7: Loss to follow up less than 5%
D8: Prospective calculation of the study type

Judgement
X High
- Moderate
+ Low

DCR according to mRECIST at 3 months follow-up:

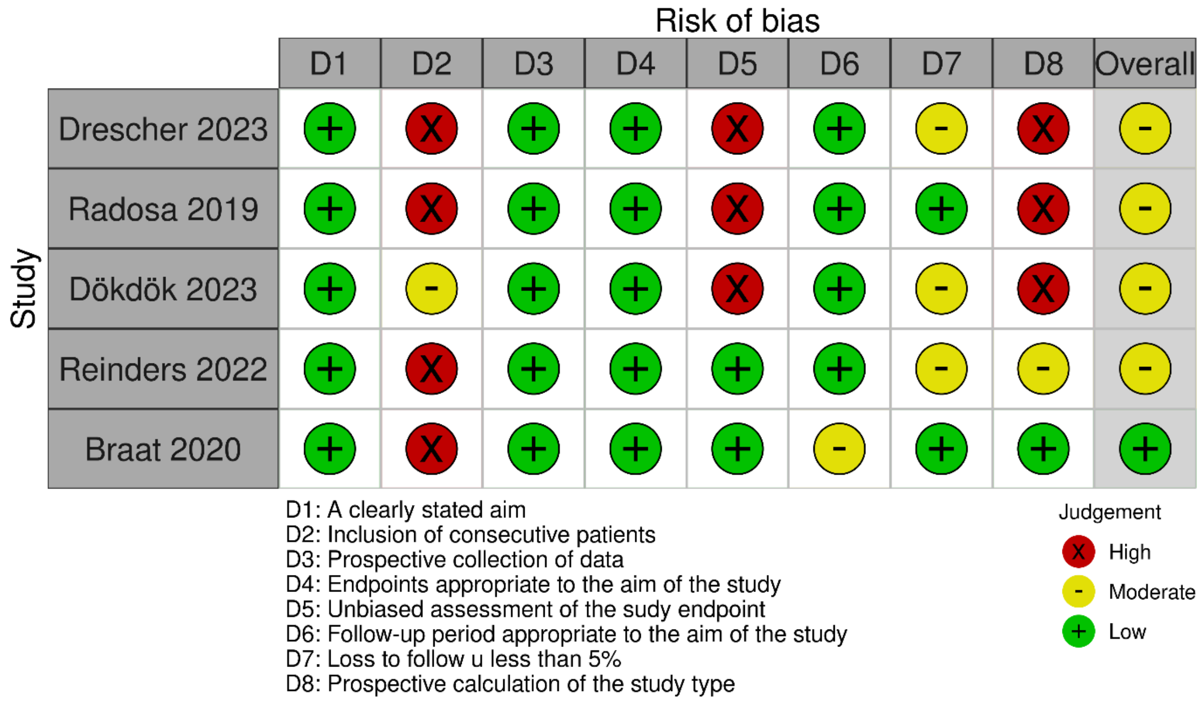

Overall survival (OS):

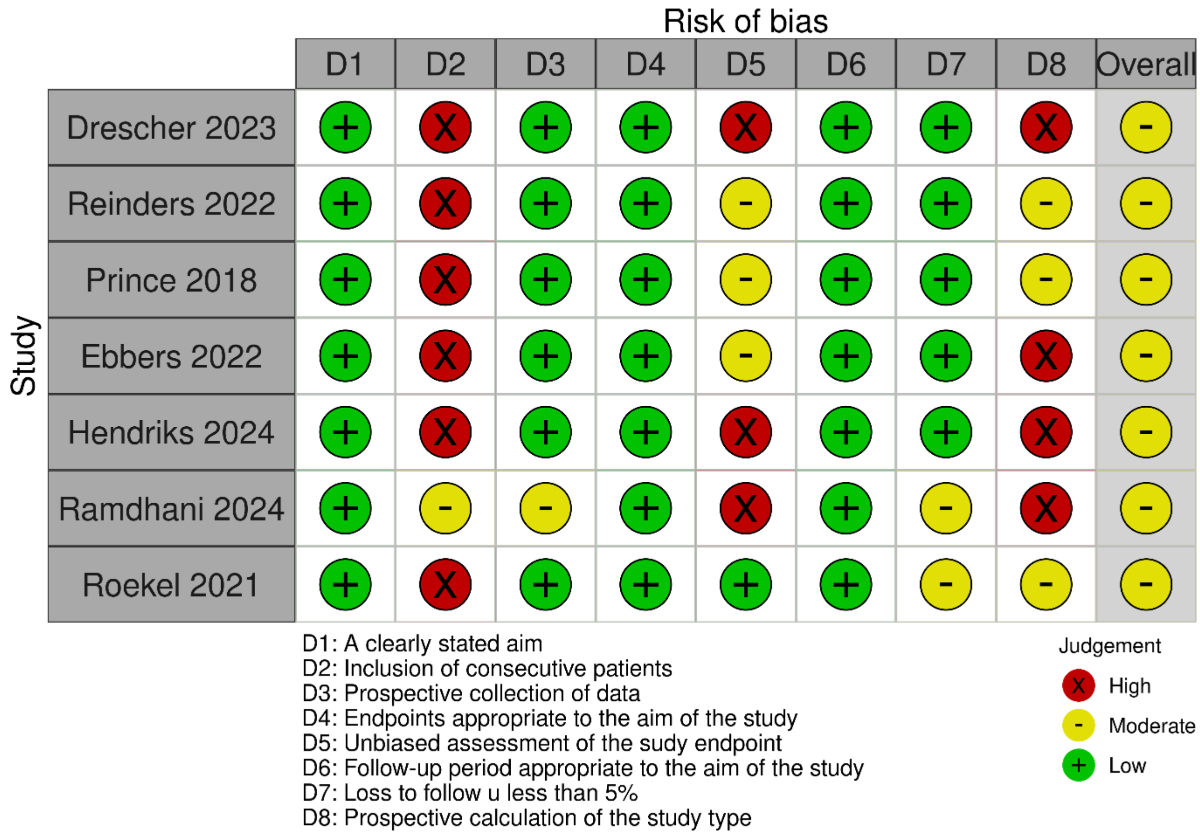

Progression-free survival (PFS):

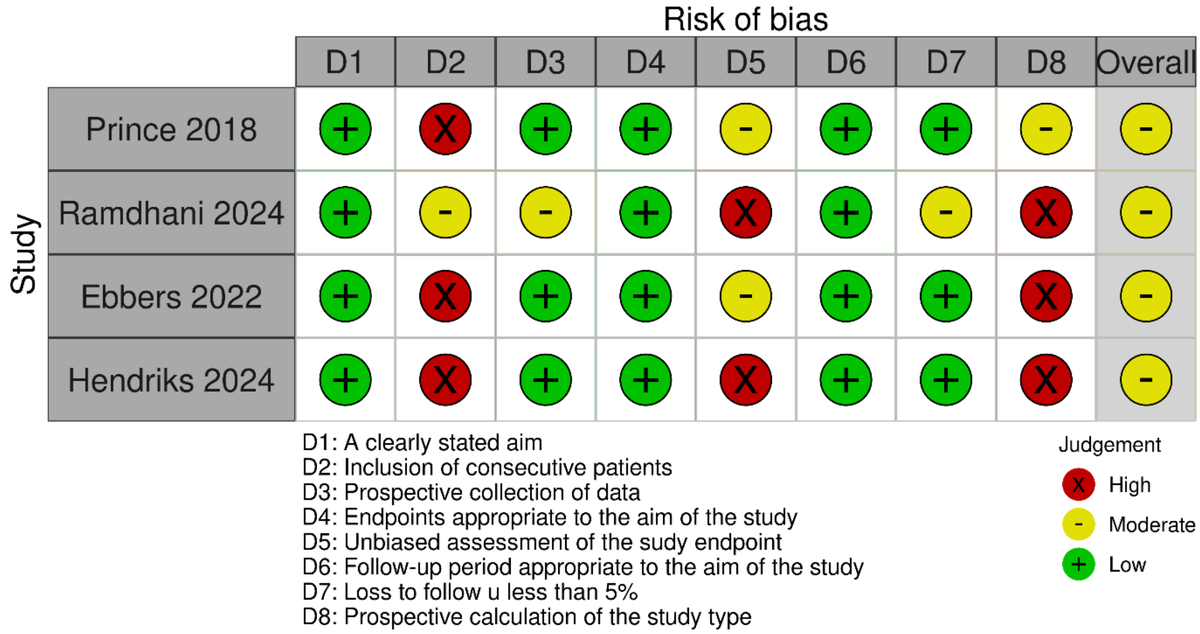

Clinical adverse events according to CTCAE:

Risk of bias

Study	D1	D2	D3	D4	D5	D6	D7	D8	Overall
Ramdhani 2024	+	-	-	+	X	+	-	X	-
Reinders 2022	+	X	+	+	+	+	+	+	+
Drescher 2023	+	X	+	+	X	+	+	X	-
Radosa 2019	+	X	+	+	X	+	+	X	-
Dökdök 2023	+	-	+	+	X	+	-	X	-
Roosen 2022	+	X	+	+	X	+	+	X	-
Smits 2012	+	X	+	+	+	+	+	X	-
Prince 2018	+	X	+	+	+	+	+	-	+
Braat 2020	+	X	+	+	+	+	+	-	+
Hendriks 2024	+	X	+	+	X	+	-	-	-

D1: A clearly stated aim
D2: Inclusion of consecutive patients
D3: Prospective collection of data
D4: Endpoints appropriate to the aim of the study
D5: Unbiased assessment of the sudy endpoint
D6: Follow-up period appropriate to the aim of the study
D7: Loss to follow u less than 5%
D8: Prospective calculation of the study type

Judgement
X High
- Moderate
+ Low

Laboratory adverse events according to CTCAE:

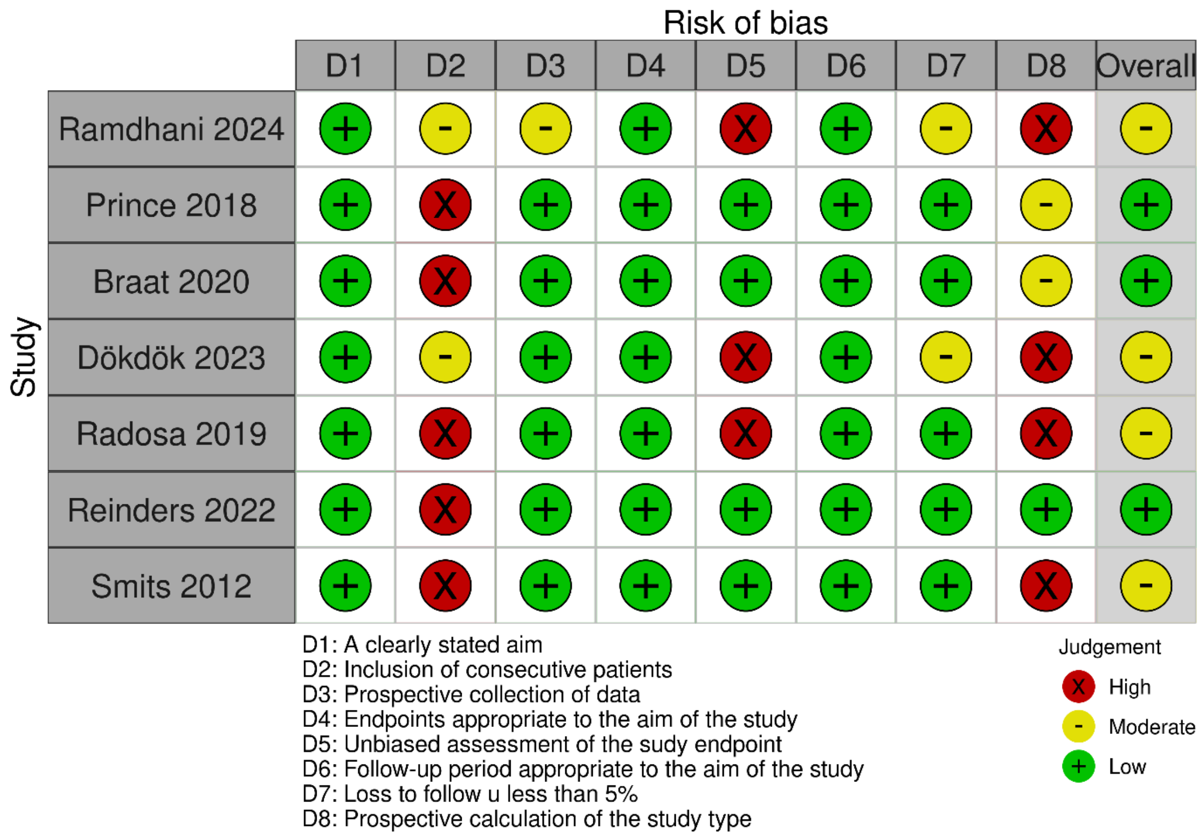

Tumor absorbed dose:

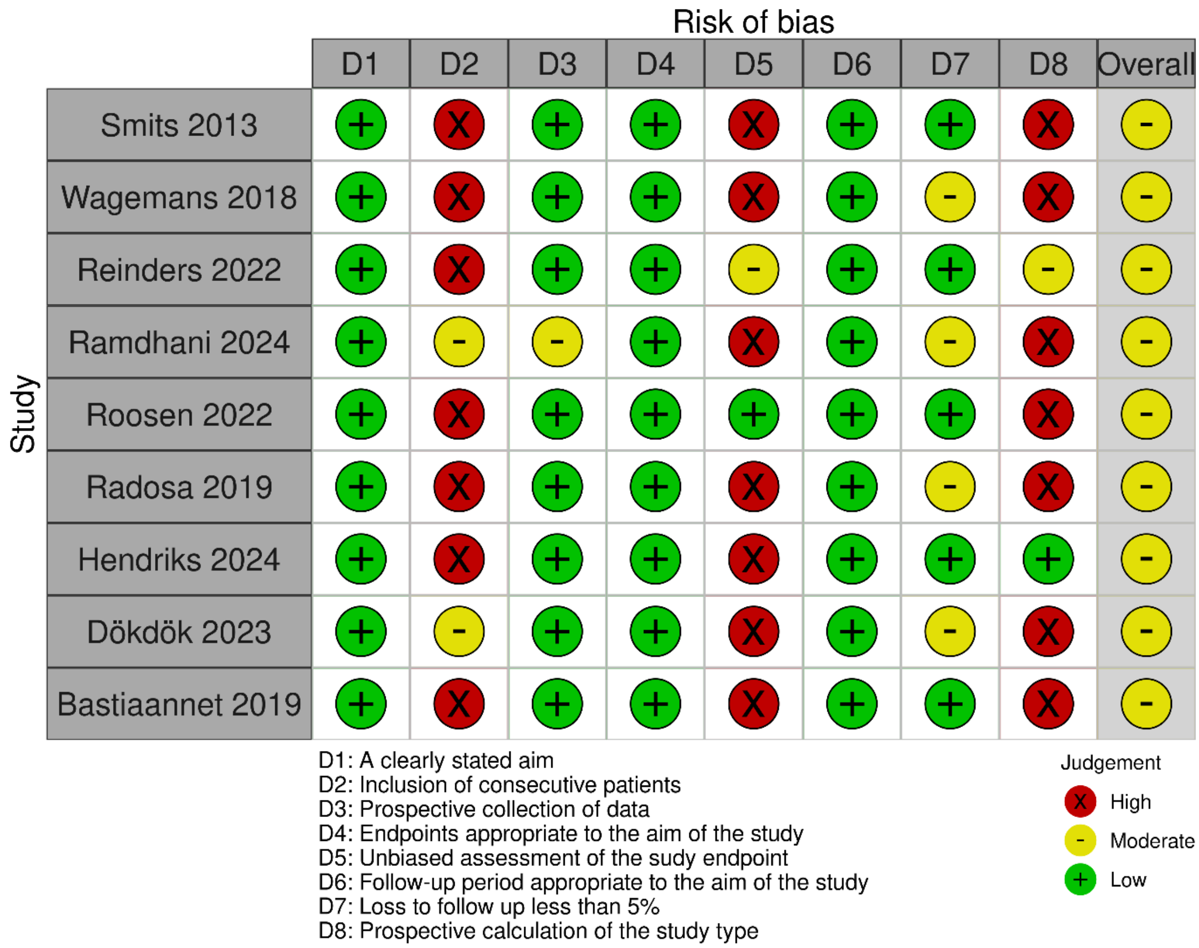

Healthy liver absorbed dose:

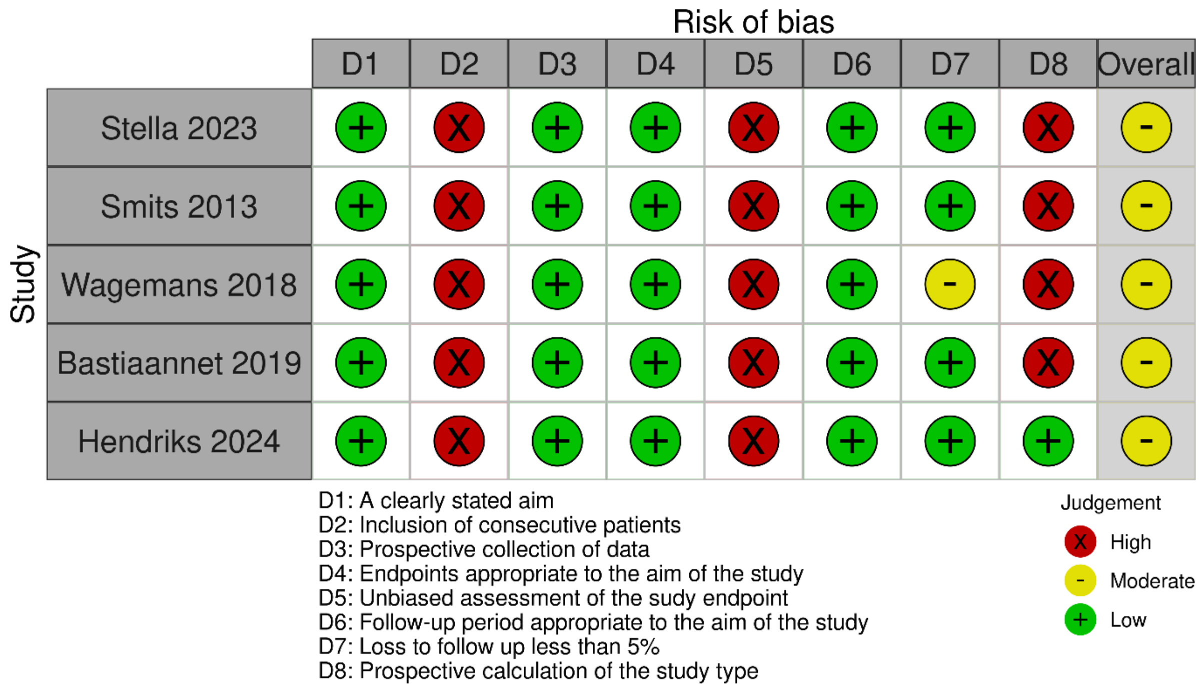

Supplement: Supplementary file 1 [file cancers-17-01841-s001.zip › Supplementary material 4_Risk of bias assessment.pdf]
