# Supplementary material for: Holmium-166 Radioembolization Is a Safe and Effective Locoregional Treatment for Primary and Secondary Liver Tumors: A Systematic Review and Meta-Analysis
Source: Cancers (Basel). 2025 May 31;17(11):1841. doi: 10.3390/cancers17111841 (PMC12153601; doi:10.3390/cancers17111841)
Supplement: Supplementary file 1 [file cancers-17-01841-s001.zip › Supplementary material 5_Prior and concurrent therapies.pdf]

Prior- and concurrent therapies with Ho-166-TARE:

First author, publication date	Total number of patients	Treatments before Ho-166-TARE	Concurrent treatments with Ho-TARE	Number of patients receiving therapy
**Braat 2020**	**30**	Peptide receptor radionuclide therapy (PRRT) with ^{177}Lu-DOTATATE		30
		Right-sided hepatectomy		1
		Bland embolisation		2
		Resection of primary tumor		12
		Somatostatin analogues		22
		Sunitinib		3
		Everolimus		2
		External-beam radiotherapy to primary site		2
			Somatostatin analogues	19
**Bastiaannet 2019**	**36**	Locoregional (liver)		8
		Systemic		34
		None		2
			None	36
**Dökdök 2023**	**9**	Chemotherapy		9
		Targeted therapy		3
		Hormon receptor therapy		1
		90Y TARE		1
		RFA		4
		TACE		3
		Right lobe metastasectomy		1
			None	9
**Drescher 2023**	**20**	None		8
		Liver surgery (resection)		4
		90Y TARE		2
		TACE		1
		Percutaneous radiation therapy		1
		Systemic therapy		5
			Systemic therapy	3
**Ebbers 2022**	**31**	Peptide receptor radionuclide therapy (PRRT) with ^{177}Lu-DOTATATE		31
			None	31

**Prince 2018**	**38**	Systemic therapy		38
		Chemotherapy		23
			None	38
**Radosa 2019**	**9**	Resection		6
		TACE		2
		None		3
			None	9
**Reinders 2022**	**31**	Resection		4
		Ablation		4
		TACE		1
		None		26
			None	31
**Roosen 2022**	**6**	Systemic therapy		4
		None		2
			None	6
**Smits 2012**	**15**	Systemic treatments		11
		Locoregional treatments		5
			None	15
**Roekel 2021**	**40**	External-beam radiation therapy		2
		Metastasectomy		5
		RFA		3
		Systemic		40
			None	40
**Smits 2013**	**15**		None	15
**Stella 2023**	**31**	Systemic		31
			None	31
**Wagemans 2024**	**7**	Chemotherapy		5
		Locoregional		1
		None		1
			None	7
**Hendriks 2024**	**12**	RFA		12
			None	12
**Ramdhani 2024**	**29**	Somatostatin analogues		23
		PRRT		17
		Primary tumor resection		13
		Chemotherapy		7
		Hepatic surgery		4
		RFA		2
		External-beam radiotherapy		2
		Ablation		2
		Bland embolization		1
		90Y TARE		2
			None	29

Supplementary Table 1 Summary of prior and concurrent therapies in patients treated with Ho-166-TARE across the included studies. PRRT=Peptide receptor radionuclide therapy. TARE=Transarterial radioembolisation. RFA=Radiofrequency ablation. TACE=Trasnarterial chemoembolisation.
