# Supplementary material for: Holmium-166 Radioembolization Is a Safe and Effective Locoregional Treatment for Primary and Secondary Liver Tumors: A Systematic Review and Meta-Analysis
Source: Cancers (Basel). 2025 May 31;17(11):1841. doi: 10.3390/cancers17111841 (PMC12153601; doi:10.3390/cancers17111841)
Supplement: Supplementary file 1 [file cancers-17-01841-s001.zip › Supplementary material 6_Tumor extent, primary tumor type or origin.pdf]

Tumor extents and primary tumor type or origin:

First Author, publication date	Primary tumor type or origin	Tumor size (mm)	Tumor volume (cm^3/ml)	Tumor burden (%)
Braat et al. 2020	• Functioning neuroendocrine neoplasms (9/30)<br>• Pancreas(9/30)<br>• Ileum or jejunum (9/30)<br>• Unknown (5/30)<br>• Colon/coecum/rectum (4/30)<br>• Bronchus/lung (3/30)	na	na	<25 (22/30)<br><br>25-50 (6/30)<br><br>50-70 (2/30)
Bastiaannet et al. 2019	• Colorectal (21/36)<br>• Breast (4/36)<br>• Cholangiocarcinoma (4/36)<br>• Uveal melanoma (4/36)<br>• Neuroendocrine neoplasm (1/36)<br>• Pancreas (1/36)<br>• Thymoma (1/36)	na	5-1993 (median: 171)	na
Dökdök et al. 2023	• Ovary (1/9)<br>• Colon (2/9)<br>• Gasrtic (1/9)<br>• Pancreas (2/9)<br>• Uveal melanoma (1/9)<br>• Breast (1/9)<br>• Cholangiocarcinoma (1/9)	10-123 (mean: 33)	na	na
Drescher et al. 2023	• HCC (14/20)<br>• Colorectal (4/20)<br>• ICC (1/20)<br>• Haemangioendothelioma (1/20)	na	17-730 (median: 127; mean: 194,3 ml ± 188)	2-58 (median: 6; mean: 15,2 ± 13,4)
Ebbers et al. 2022	• Pancreas (10/31)<br>• Small intestine (8/31)<br>• Colorectal (4/31)<br>• Lung (3/31)<br>• Unknown (6/31)	na	na	3,1-22,5 (median: 6,9)
Prince et al. 2018	• Colorectal (23/38)<br>• Breast (4/38)<br>• Cholangiocarcinoma (4/38)<br>• Neuroendocrine tumors (2/38)<br>• Uveal melanoma (2/38)<br>• Pancreas (1/38)<br>• Gastric (1/38)<br>• Thymoma (1/38)	na	na	<25 (30/38)<br><br>25-50 (6/38)<br><br>50< (2/38)
Radosa et al. 2019	• HCC (9/9)	na	na	na
Reinders et al. 2022	• HCC (31/31)	15-195 (median: 56)	na	0,5-46,8 (median: 9,3)

Roosen et al. 2022	• Breast (2/6)<br>• Cholangiocarcinoma (1/6)<br>• HCC (1/6)<br>• Colorectal (1/6)	na	na	4,3-68,8 (median: 36,2)
Smits et al. 2012	• Ocular melanoma (6/15)<br>• Colorectal (6/15)<br>• Cholangiocarcinoma (2/15)<br>• Breast (1/15)	na	na	2-52 (median: 14)<br><br><25 (10/15)<br><br>25-50 (4/15)<br><br>>50 (1/15)
Roekel et al. 2021	• Colorectal (40/40)	na	26 -1446 (median: 320)	na
Smits et al. 2013	• Ocular melanoma (6/15)<br>• Colorectal (6/15)<br>• Cholangiocarcinoma (2/15)<br>• Breast (1/15)	na	na	2-52 (median: 14)<br><br><25 (10/15)<br><br>25-50 (4/15)<br><br>>50 (1/15)
Stella et al. 2023	• Pancreas (10/31)<br>• Small intestine (8/31)<br>• Colorectal (4/31)<br>• Lung (3/31)<br>• Unknown (6/31)	na	na	3,1-22,5 (median: 6,9)
Wagemans et al. 2024	• ICC (7/7)	na	16-2655 (median: 146)	na
Hendriks et al. 2024	• HCC (12/12)	21-40 (median: 27)	na	na
Ramdhani et al. 2024	• Small bowel (16/29)<br>• Pancreas (7/29)<br>• Lung (4/29)<br>• Gastric (1/29)<br>• Unknown (1/29)	10-127 (median: 27)	3-2470 (median: 272)	0,2-69,6 (median: 14)

Supplementary Table 2 Summary of primary tumor types and tumor extent as reported in the included articles, based on size, volume, or tumor burden. HCC=hepatocellular carcinoma. ICC=intrahepatic cholangiocarcinoma.
