# Supplementary material for: Holmium-166 Radioembolization Is a Safe and Effective Locoregional Treatment for Primary and Secondary Liver Tumors: A Systematic Review and Meta-Analysis
Source: Cancers (Basel). 2025 May 31;17(11):1841. doi: 10.3390/cancers17111841 (PMC12153601; doi:10.3390/cancers17111841)

Funnel plots (to assess symmetry and reporting bias) and Baujat plots (to detect the potential sources of heterogeneity) for the primary and secondary outcomes

Overall DCR at 3 months:

Funnel plot:

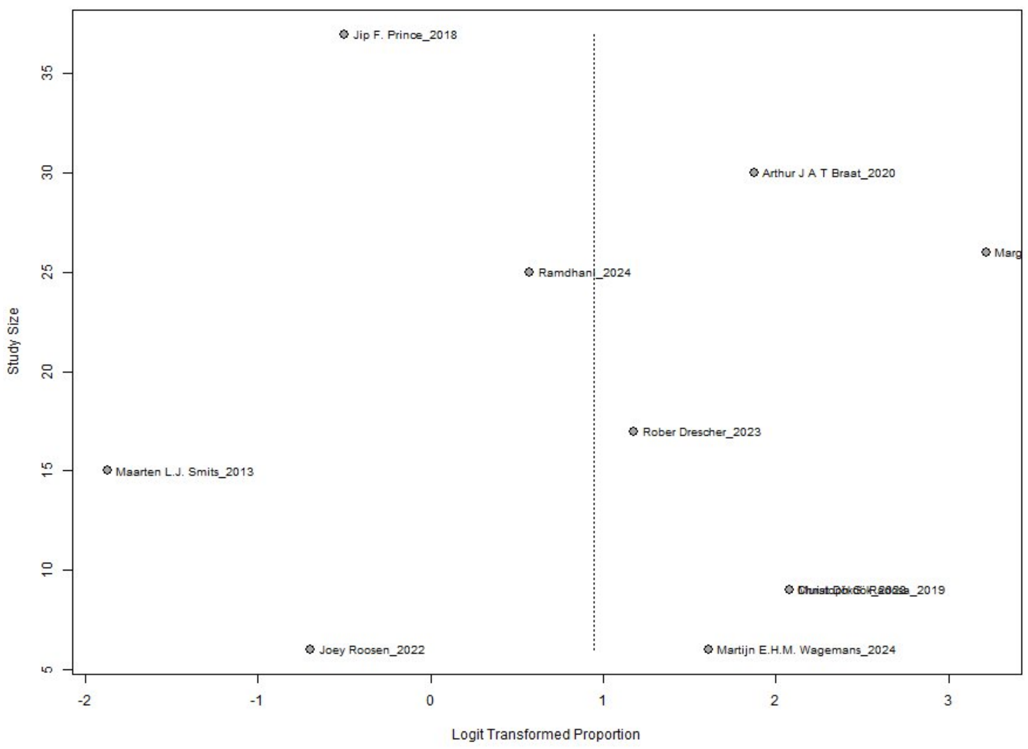

Baujat plot:

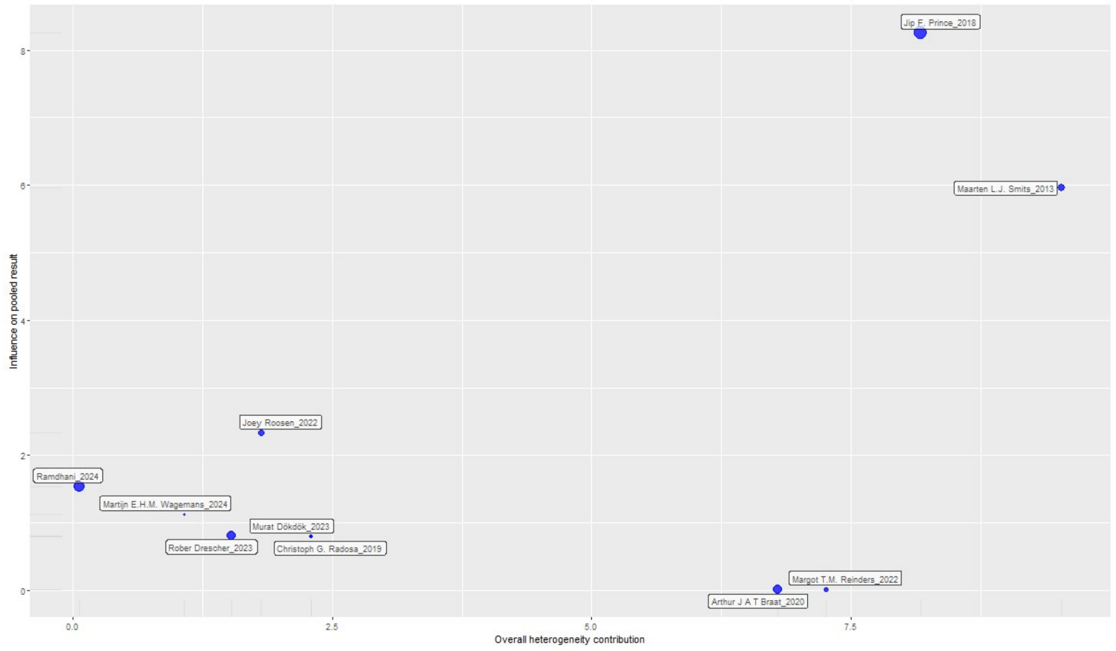

DCR according to RECIST 1.1 at 3 months:

Funnel plot:

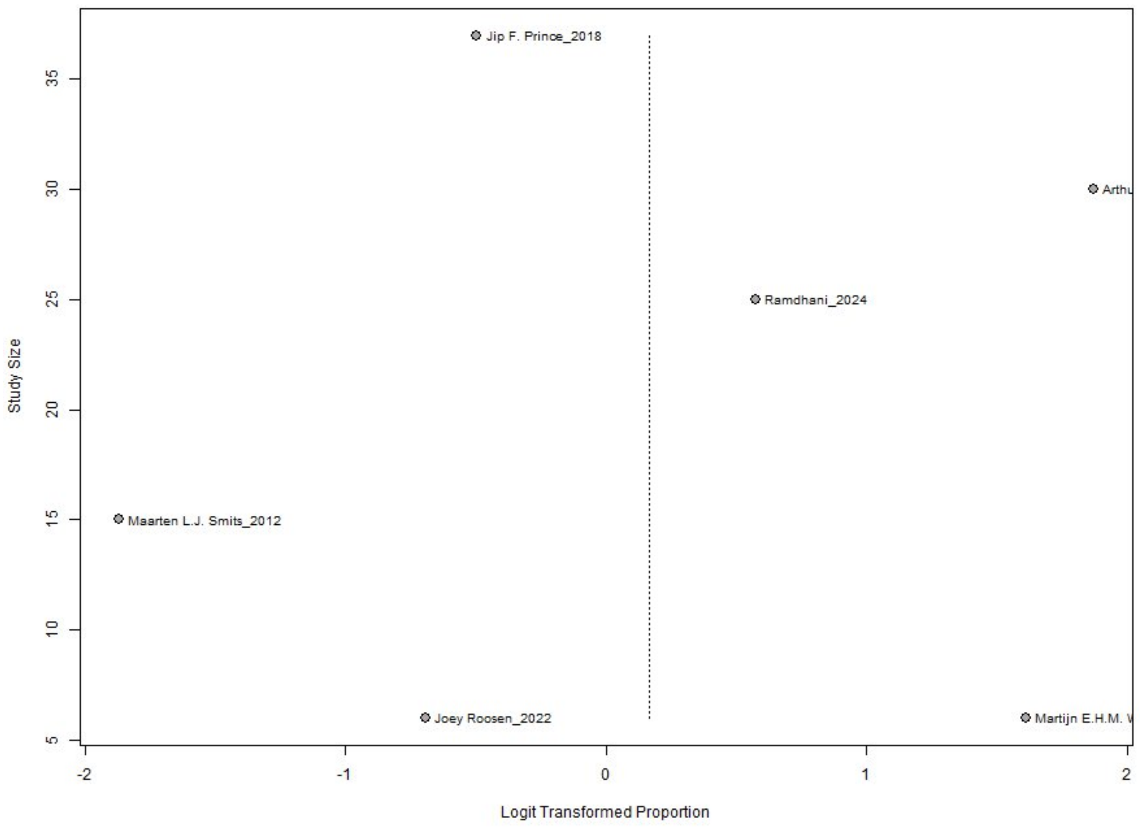

Baujat plot:

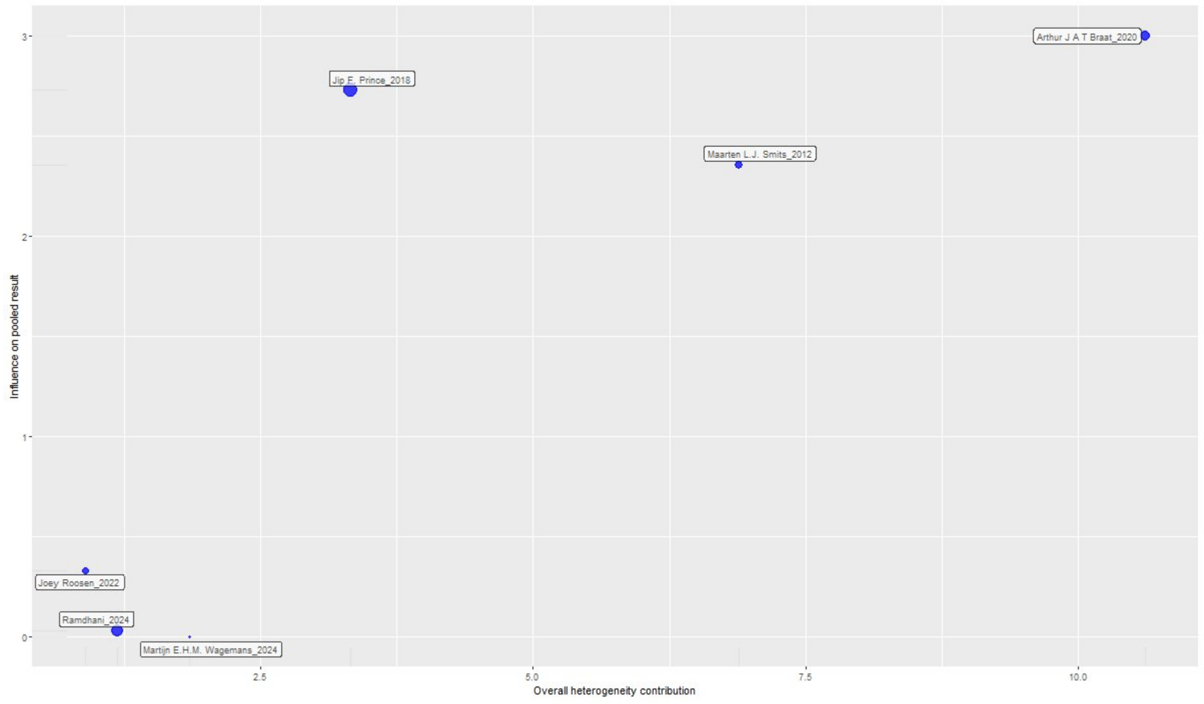

DCR according to mRECIST at 3 months:

Funnel plot:

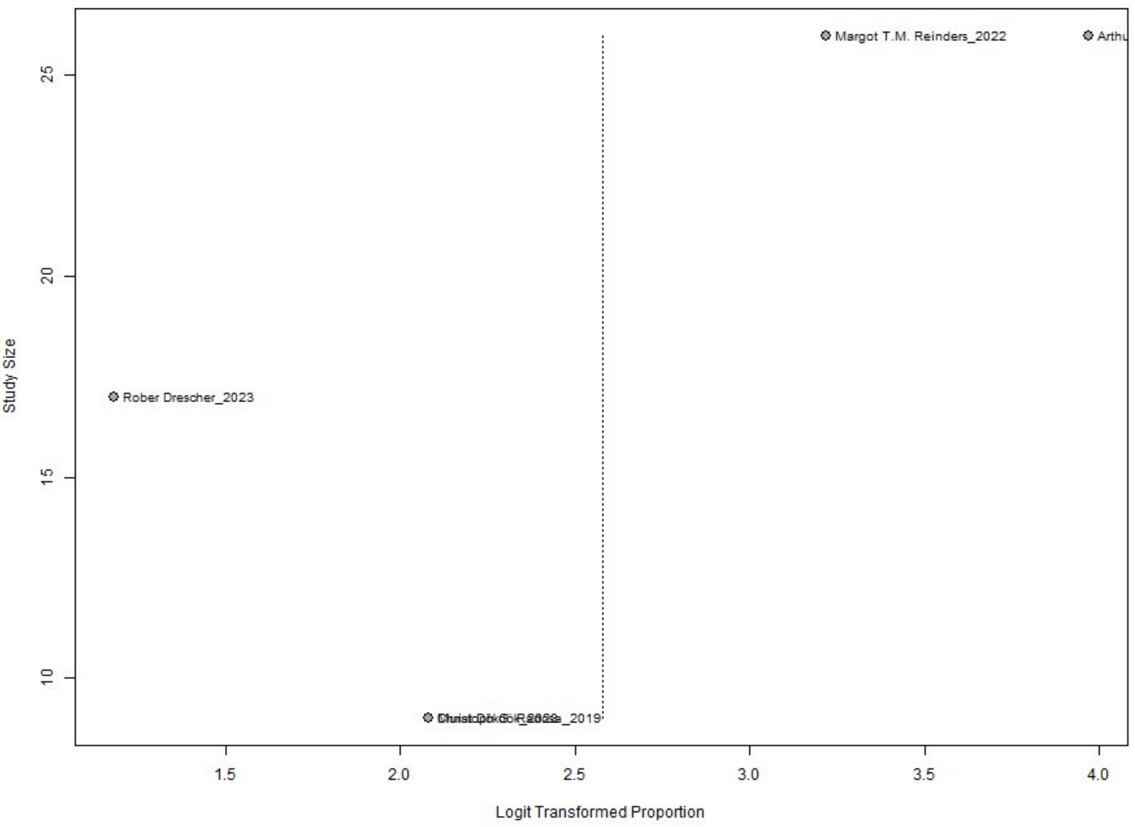

Baujat plot:

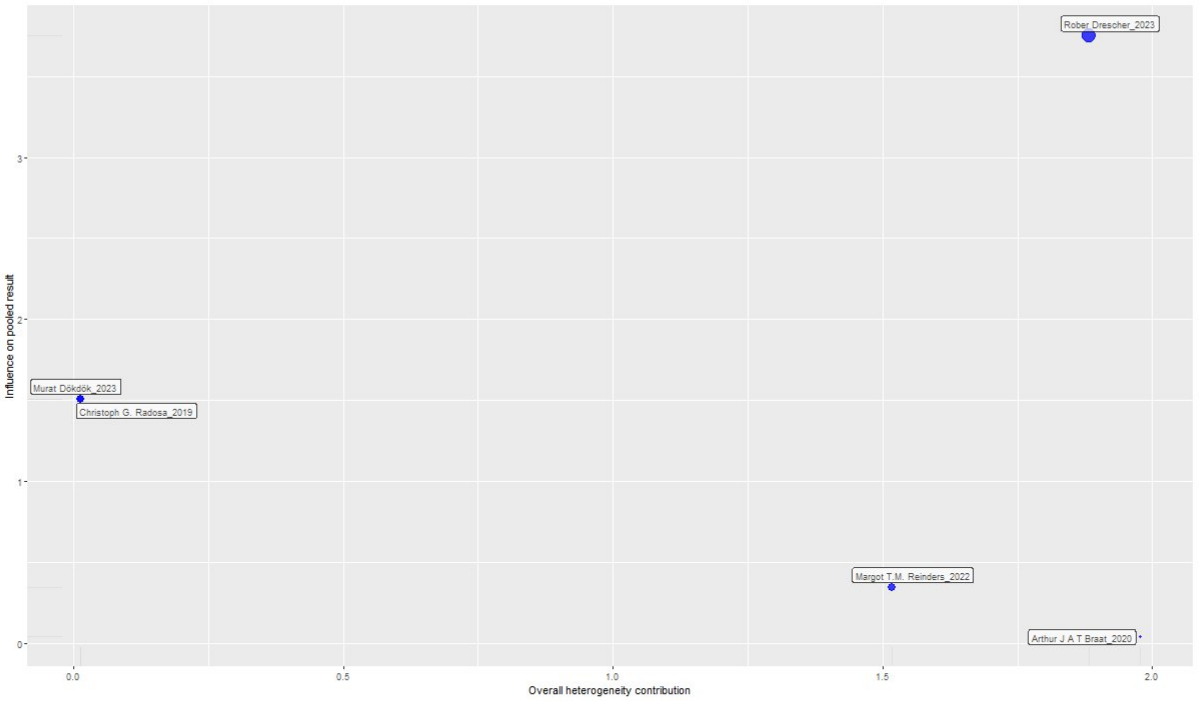

Severe GGT increase:

Funnel plot:

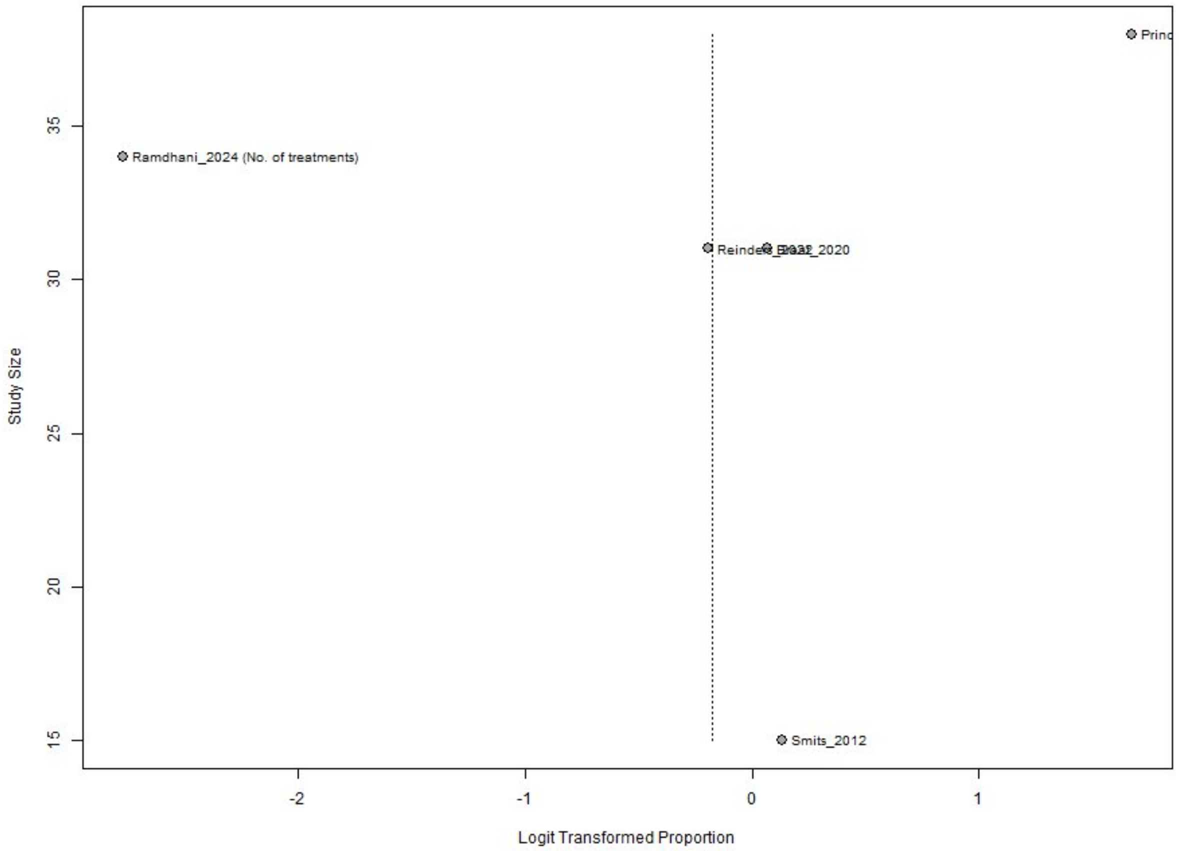

Baujat plot:

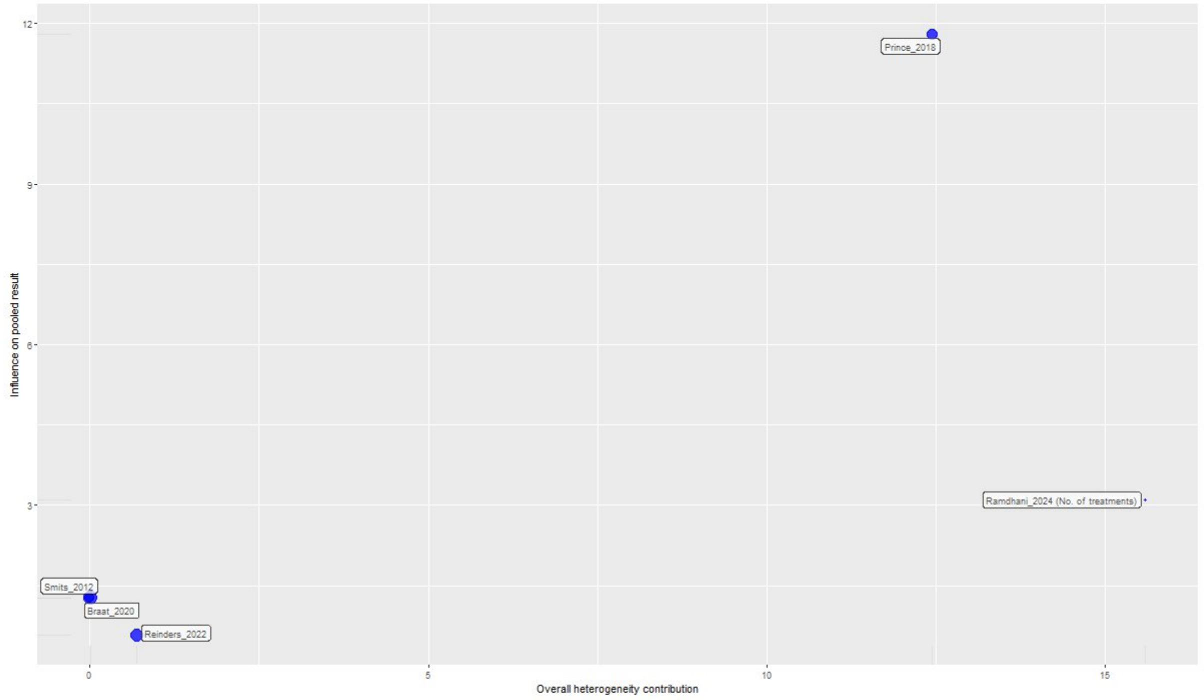

Severe lymphocytopenia:

Funnel plot:

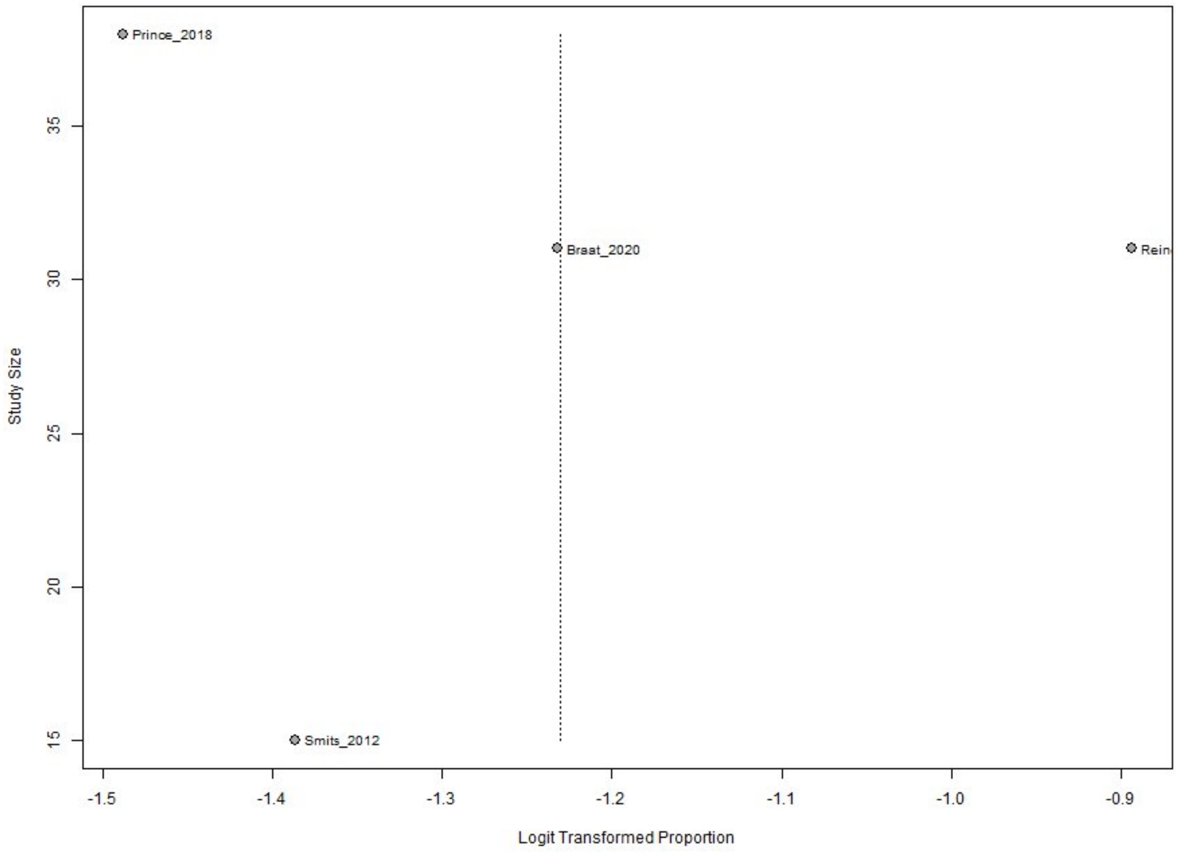

Baujat plot:

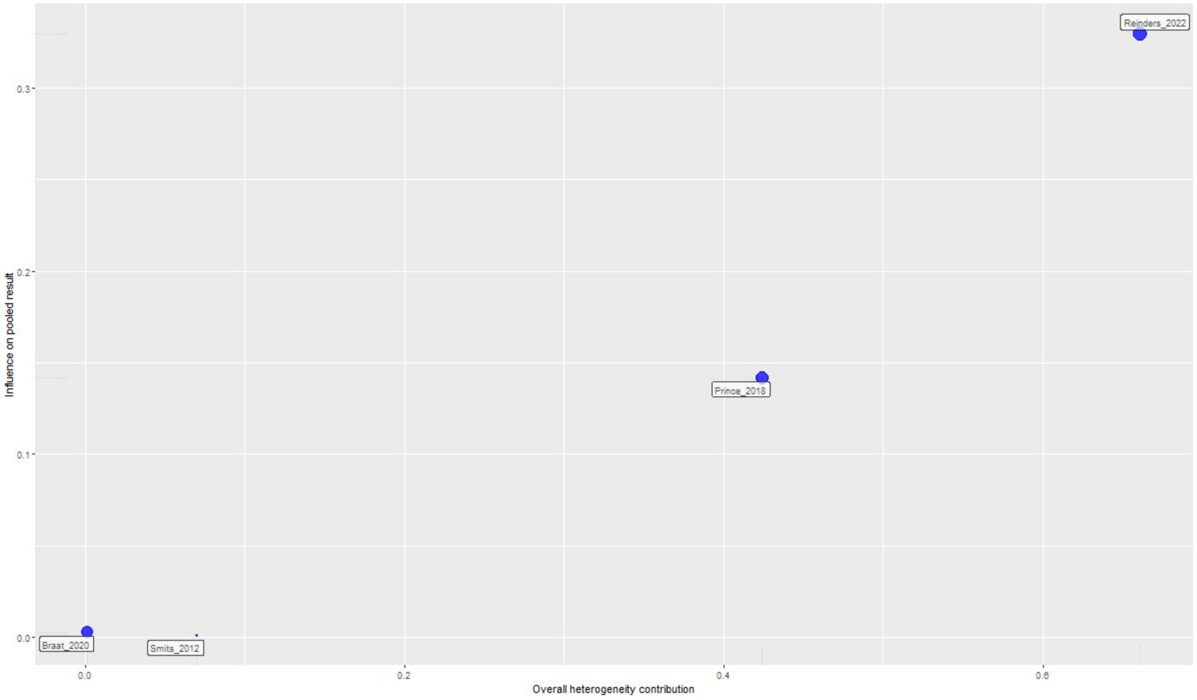

Tumor-absorbed dose:

Funnel plot:

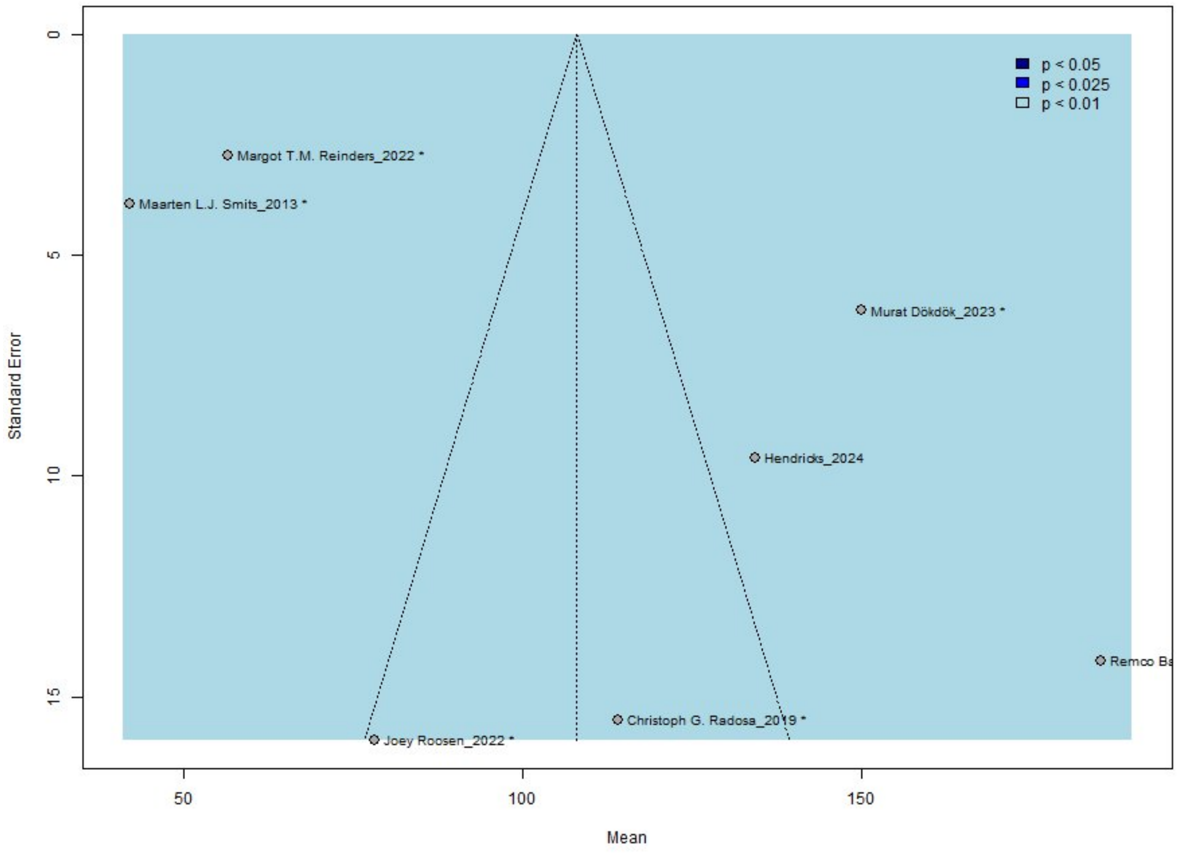

Baujat plot:

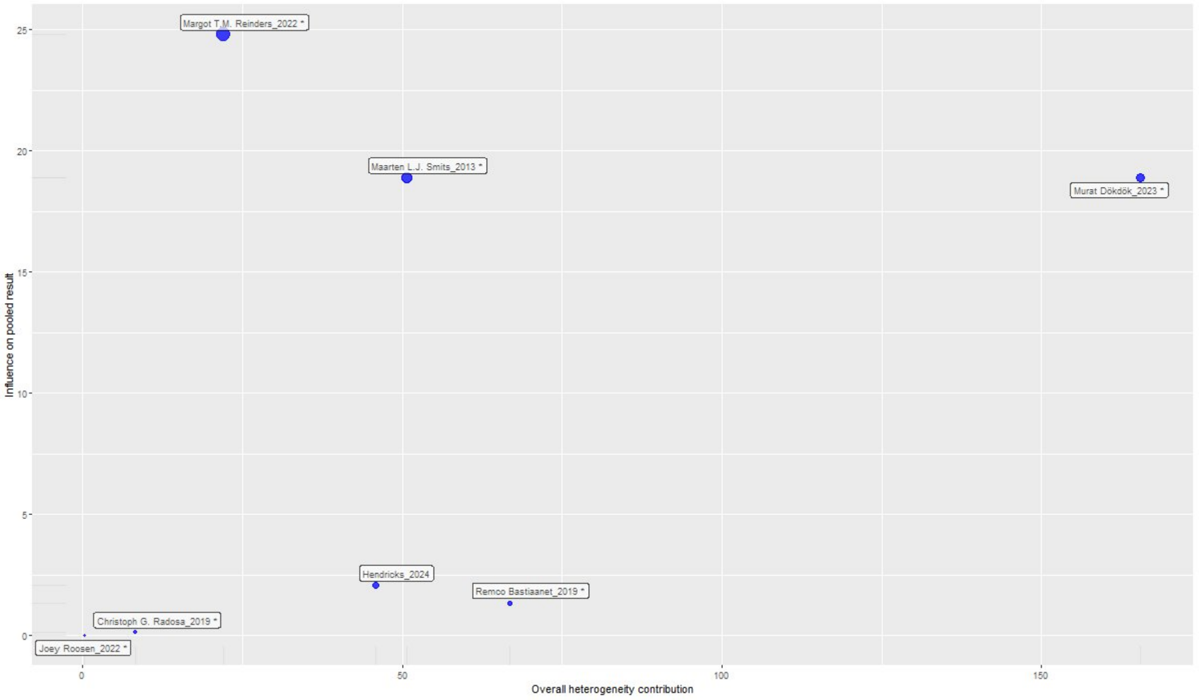

Healthy liver-absorbed dose:

Funnel plot:

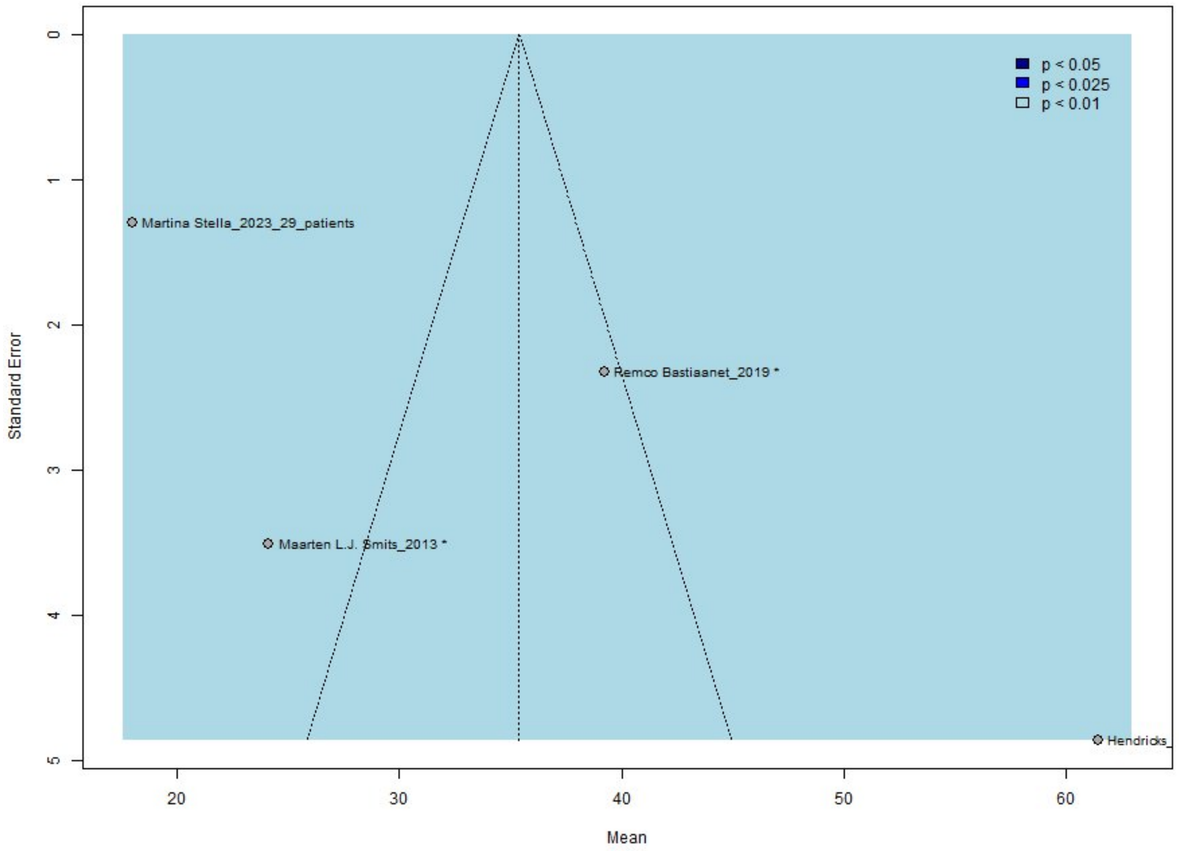

Baujat plot:

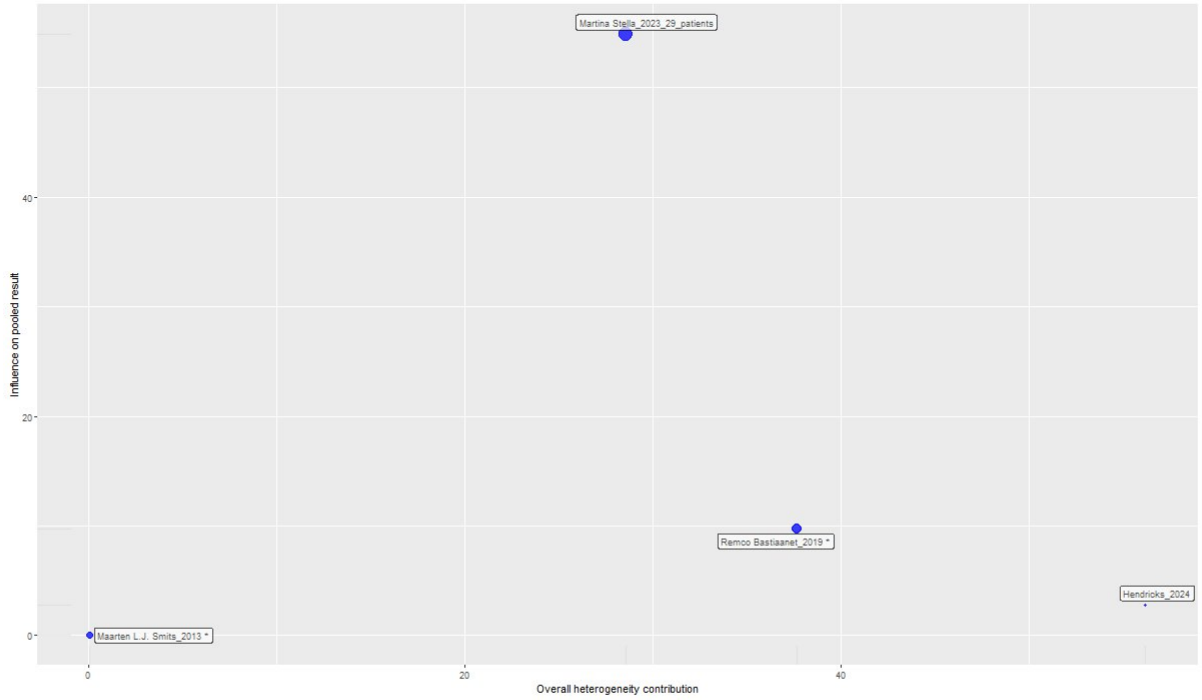

Supplement: Supplementary file 1 [file cancers-17-01841-s001.zip › Supplementary material 7_Funnel plots and Baujat plots.pdf]
