# Supplementary material for: Holmium-166 Radioembolization Is a Safe and Effective Locoregional Treatment for Primary and Secondary Liver Tumors: A Systematic Review and Meta-Analysis
Source: Cancers (Basel). 2025 May 31;17(11):1841. doi: 10.3390/cancers17111841 (PMC12153601; doi:10.3390/cancers17111841)
Supplement: Supplementary file 1 [file cancers-17-01841-s001.zip › Supplementary material 8_Literature string.pdf]

Clinical question:

In patients with a liver tumor, is transarterial radioembolization (TARE) using holmium isotope (^{166}Ho) a safe and efficient way of transarterial treatment?

Framework:

P: Patients with primary or secondary liver tumors undergoing radioembolization

E: TARE using holmium isotopes (^{166}Ho)

O: Tumor response rate (primary endpoint), overall survival, toxicity (secondary endpoints)

Search Key:

(liver* **OR** "hepatic" **OR** ("liver" AND tumo*) **OR** ("liver" AND neoplasm*) **OR** ("liver" AND "cancer") **OR** ("hepatocellular" AND "carcinoma") **OR** "hepatocarcinoma" **OR** ("hepatic" AND "cancer") **OR** "HCC" **OR** ("cholangiocellular" AND "carcinoma") **OR** cholangiocarcinoma* **OR** "ICC" **OR** ("liver" AND metasta*))

AND

("TARE" OR radioemboli* **OR** "transarterial" **OR** "transarterially" **OR** ("transarterial" AND radioemboli*))

AND

("holmium" **OR** ("ho" AND "166") **OR** "166ho")

Domain 1 refers to: Patient population

Domain 2 refers to: Intervention

Domain 2 refers to: Holmium-166

Time of search:

2023.11.01.

Updated: 2024.06.05.

Name of database, settings, and the number of results

PubMed: Advanced, automatic term translation off (quotation marks), no filter. **84** (2023.11.01.) and **96** (2024.06.05.) results

EMBASE: Advanced, automatic term translation off, every setting in mapping is unchecked, no filter, Title-Abstract-Keyword searched. **129** (2023.11.01.) and **191** (2024.06.05.) results

Cochrane Library: Advanced, automatic term translation off, Title-Abstract-Keyword searched, no filter, results in trials. **8** (2023.11.01.) and **8** (2024.06.05.) results

Scopus: Advanced, automatic term translation off, Title-Abstract-Keyword searched, no filter. **117** (2023.11.01.) and **136** (2024.06.05.) results

Web of Science: Advanced, automatic term translation off, no filter, Exact Search used, **113** (2023.11.01.) and **129** (2024.06.05.) results

Explanation:

Truncation: liver/livers/liver’s; tumor/tumour; neoplasm/neoplasms; cholangiocarcinoma/cholangiocarcinomas; metastasis/metastases; radioembolization/radioembolisation

Quotation marks: to turn off term translation
