# Supplementary material for: Holmium-166 Radioembolization Is a Safe and Effective Locoregional Treatment for Primary and Secondary Liver Tumors: A Systematic Review and Meta-Analysis
Source: Cancers (Basel). 2025 May 31;17(11):1841. doi: 10.3390/cancers17111841 (PMC12153601; doi:10.3390/cancers17111841)

<u>Forest plots for overall responders (complete response + partial response):</u>

Study	Events	Total	Proportion	95%-CI
Smits 2013	1	15	0.07	[0.00; 0.32]
Prince 2018	5	37	0.14	[0.05; 0.29]
Wagemans 2024	1	6	0.17	[0.00; 0.64]
Roosen 2022	2	6	0.33	[0.04; 0.78]
Braat 2020	12	30	0.40	[0.23; 0.59]
Reinders 2022	14	26	0.54	[0.33; 0.73]
Radosa 2019	5	9	0.56	[0.21; 0.86]
Dökdök 2023	5	9	0.56	[0.21; 0.86]
Ramdhani 2024	14	25	0.56	[0.35; 0.76]
Drescher 2023	11	17	0.65	[0.38; 0.86]
**Random effects model**	**70**	**180**	**0.38**	**[0.23; 0.56]**
**Prediction interval**				**[0.08; 0.82]**

Heterogeneity: I^2 = 64% [28%; 82%], τ^2 = 0.65, p = 0.003

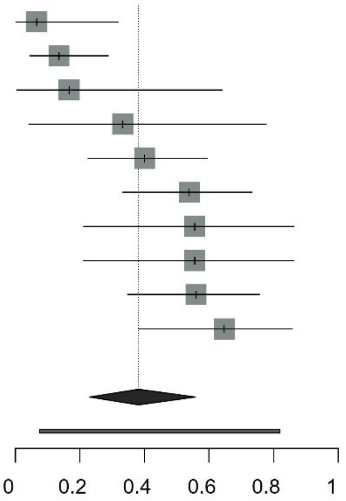

<u>Forest plot for responders according to RECIST 1.1:</u>

Study	Events	Total	Proportion	95%-CI
Smits 2012	1	15	0.07	[0.00; 0.32]
Prince 2018	5	37	0.14	[0.05; 0.29]
Wagemans 2024	1	6	0.17	[0.00; 0.64]
Roosen 2022	2	6	0.33	[0.04; 0.78]
Braat 2020	12	30	0.40	[0.23; 0.59]
Ramdhani 2024	14	25	0.56	[0.35; 0.76]
**Random effects model**	**35**	**119**	**0.26**	**[0.10; 0.51]**
**Prediction interval**				**[0.03; 0.82]**

Heterogeneity: I^2 = 69% [27%; 87%], τ^2 = 0.67, p = 0.007

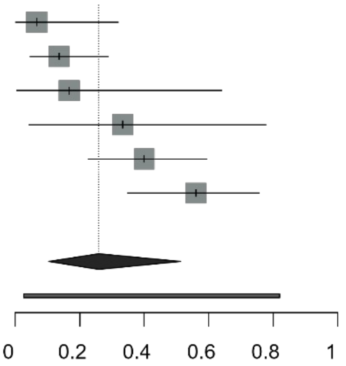

<u>Forest plot for responders according to mRECIST:</u>

Study	Events	Total	Proportion	95%-CI
Reinders 2022	14	26	0.54	[0.33; 0.73]
Radosa 2019	5	9	0.56	[0.21; 0.86]
Dökdök 2023	5	9	0.56	[0.21; 0.86]
Drescher 2023	11	17	0.65	[0.38; 0.86]
Braat 2020	18	26	0.69	[0.48; 0.86]
**Random effects model**	**53**	**87**	**0.61**	**[0.46; 0.74]**
**Prediction interval**				**[0.44; 0.76]**

Heterogeneity: I^2 = 0% [0%; 79%], τ^2 = 0, p = 0.808

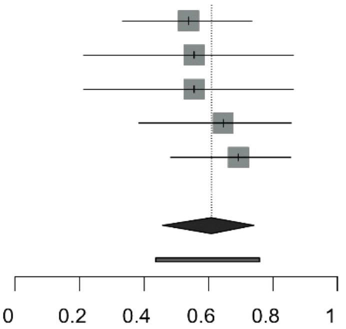

Supplement: Supplementary file 1 [file cancers-17-01841-s001.zip › Supplementary material 1_Responders.pdf]
